# Thymic Epithelium Abnormalities in DiGeorge and Down Syndrome Patients Contribute to Dysregulation in T Cell Development

**DOI:** 10.3389/fimmu.2019.00447

**Published:** 2019-03-15

**Authors:** Genni Enza Marcovecchio, Ileana Bortolomai, Francesca Ferrua, Elena Fontana, Luisa Imberti, Erika Conforti, Donato Amodio, Sonia Bergante, Giulia Macchiarulo, Veronica D'Oria, Francesca Conti, Silvia Di Cesare, Georgia Fousteri, Adriano Carotti, Alessandro Giamberti, Pietro Luigi Poliani, Luigi D. Notarangelo, Caterina Cancrini, Anna Villa, Marita Bosticardo

**Affiliations:** ^1^Division of Regenerative Medicine, Stem Cells and Gene Therapy, Telethon Institute for Gene Therapy (SR-Tiget), IRCCS San Raffaele Scientific Institute, Milan, Italy; ^2^Department of Systems Medicine, University of Rome Tor Vergata, Rome, Italy; ^3^The Milan Unit, Istituto di Ricerca Genetica e Biomedica, Consiglio Nazionale delle Ricerche, Milan, Italy; ^4^Vita-Salute San Raffaele University, Milan, Italy; ^5^Pediatric Immunohematology and Bone Marrow Transplantation Unit, IRCCS San Raffaele Scientific Institute, Milan, Italy; ^6^Humanitas Clinical and Research Center, Rozzano, Milan, Italy; ^7^Laboratorio CREA (Centro di Ricerca Emato-oncologica AIL), ASST Spedali Civili of Brescia, Brescia, Italy; ^8^Department of Pediatric Cardiac Surgery, IRCCS San Donato Milanese Hospital, San Donato Milanese, Milan, Italy; ^9^University Department of Pediatrics, Bambino Gesù Children's Hospital, IRCCS, Rome, Italy; ^10^Laboratory of Stem Cells for Tissue Engineering, Istituto di Ricovero e Cura a Carattere Scientifico, Policlinico San Donato, Milan, Italy; ^11^Division of Immunology Transplantation and Infectious Diseases, Diabetes Research Institute, IRCCS San Raffaele Scientific Institute, Milan, Italy; ^12^Department of Pediatric Cardiac Surgery, IRCCS Bambino Gesú Children's Hospital, Rome, Italy; ^13^Department of Congenital Cardiac Surgery, IRCCS Policlinico San Donato, San Donato Milanese, Milan, Italy; ^14^Pathology Unit, Department of Molecular and Translational Medicine, University of Brescia, Brescia, Italy; ^15^Laboratory of Clinical Immunology and Microbiology, IDGS, DIR, NIAID, NIH, Bethesda, MD, United States

**Keywords:** thymus, DiGeorge syndrome, Down syndrome, regulatory T (Treg) cells, thymocytes

## Abstract

The thymus plays a fundamental role in establishing and maintaining central and peripheral tolerance and defects in thymic architecture or AIRE expression result in the development of autoreactive lymphocytes. Patients with partial DiGeorge Syndrome (pDGS) and Down Syndrome (DS) present alterations in size and architecture of the thymus and higher risk to develop autoimmunity. We sought to evaluate thymic architecture and thymocyte development in DGS and DS patients and to determine the extent to which thymic defects result in immune dysregulation and T cell homeostasis perturbation in these patients. Thymi from pediatric patients and age-matched controls were obtained to evaluate cortex and medullary compartments, AIRE expression and thymocyte development. In the same patients we also characterized immunophenotype of peripheral T cells. Phenotypic and functional characterization of thymic and peripheral regulatory T (Treg) cells was finally assessed. Histologic analysis revealed peculiar alterations in thymic medulla size and maturation in DGS and DS patients. Perturbed distribution of thymocytes and altered thymic output was also observed. DGS patients showed lower mature CD4^+^ and CD8^+^ T cell frequency, associated with reduced proportion and function of Tregs both in thymus and peripheral blood. DS patients showed increased frequency of single positive (SP) thymocytes and thymic Treg cells. However, Tregs isolated both from thymus and peripheral blood of DS patients showed reduced suppressive ability. Our results provide novel insights on thymic defects associated with DGS and DS and their impact on peripheral immune dysregulation. Indeed, thymic abnormalities and defect in thymocyte development, in particular in Treg cell number and function could contribute in the pathogenesis of the immunodysregulation present in pDGS and in DS patients.

## Introduction

The thymus is a specialized primary lymphoid organ involved in the development of thymocytes and has a fundamental role in establishing immune tolerance (1). It represents a crucial component of the adaptive immune system, since it is required for T-cell differentiation and repertoire selection. Moreover, it plays an important role in the control of self-tolerance through the generation of T regulatory (Treg) cells and by the negative selection of autoreactive T cells. Indeed, abnormalities in thymic compartments, due to alterations affecting either thymic epithelial cells (TEC) or T-cell development and function, lead to a breakdown of central tolerance and result in increased susceptibility to infections and autoimmune manifestations (2). In this scenario, the most common thymic stromal defect causing T-cell immunodeficiency is the 22q11.2 deletion syndrome (22q11.2DS), caused by the deletion of the long arm of chromosome 22 at position q11.2 (3, 4). This syndrome is frequently associated with DiGeorge Syndrome (DGS) (5) and in this manuscript we analyzed only DGS patients carrying the 22q11.2. In over 90% of 22q11.2DS cases the deletion is of 3 Mb and includes more than 30 different genes; the remaining patients have a smaller deletion of 1.5 Mb (6–9). It is largely demonstrated that in both cases one of the genes deleted is Tbx1, which plays an important role in thymus embryogenesis (10, 11). DGS can be associated with different degrees of T-cell deficiency ranging from normal/mild T-cell number and function, characterizing pDGS, to complete DGS (cDGS) with a T-negative severe combined immunodeficiency (SCID)-like picture (5). DGS patients are characterized by a deregulated peripheral T-cell homeostasis and a loss of Treg suppressive capacity that could compromise the integrity of T-cell immunity during adulthood leading to an increased incidence of infections and autoimmune diseases (12, 13). Another syndrome that negatively impacts on thymic functionality is the Down Syndrome (DS), which is caused by an extra copy of chromosome 21 resulting in trisomy (14). Several studies indicated alterations in the thymic stroma (15) and in the adaptive immune system in individuals with DS, with defects in both immature progenitor cells or mature peripheral lymphocytes in the peripheral blood, contributing to the development of autoimmune phenomena reported in these patients (16, 17).

Thus, DGS and DS share common immunological features, such as increased number of infections, impaired development of T cells, including natural Tregs, and impaired thymic central tolerance that may predispose to the development of autoimmunity (13, 18, 19). Limited data are available on thymic tissue impairment of patients diagnosed with DGS and DS and there is little knowledge about immune alterations and long-term consequences of their altered immune system homeostasis. Lymphopenia found in both groups may be compensated over time by a peripheral extra-thymic T-cell homeostatic proliferation (13, 19, 20), but this process may skip thymic Treg control leading to a breakdown in self-tolerance. As a result, it is not yet known whether the dysregulation of the immune system in patients affected by DGS and DS is the result of thymic alterations or quantitative/qualitative lymphocyte defects in periphery.

To clarify the role of thymic defects in the pathogenesis of autoimmune manifestations in DGS and DS patients, we collected thymic tissue and peripheral blood from both cohorts of patients. Here, we combine a detailed phenotypical analysis of T-cell maturational stages, from the early thymic progenitors to the most mature T cells in periphery, with a particular focus on the thymic compartment. A detailed analysis of thymic cortex and medulla maturation showed an altered development of the thymic epithelium with features peculiar to each syndrome, although our conclusions on the thymic stroma would need to be confirmed in a large cohort of DGS and DS patients. Our data demonstrate a perturbed distribution of thymocytes and an altered cortical/medullary thymic organization contributing to abnormalities in central and peripheral immunity. In summary, our study of T-cell development in thymus and periphery, at phenotypical and functional levels, provides novel evidence on the role of thymic defects on the pathophysiology of immune dysregulation in DGS and DS patients.

## Methods

### Patients

Thymus and peripheral blood of HD, DGS and DS patients were obtained according to The Code of Ethics of the World Medical Association (Declaration of Helsinki) with the approval of the local Medical Ethical Committees of the Bambino Gesù Children Hospital (DGS_Project_OPBG_2015), Policlinico San Donato (TIGET07) and San Raffaele Scientific Institute. For patients' enrollment we established inclusion and exclusion criteria and we admitted in the study patients between 0 and 6 years old, affected by a congenital heart disease and candidate to a first cardiac surgery with sternotomy. Patients who did not fulfill these criteria or were affected by a known infectious disease and characterized by a previous history of chemo/radiotherapy were excluded from the study. In our study we enrolled 26 Healthy Donors (HD), 10 DGS and 9 DS patients. To increase the number of DGS patients included in the study, we collected peripheral blood samples from the Pediatric Department of San Raffaele Hospital. Samples were obtained according to the Helsinki Declaration with the approval of the local Medical Ethical Committees of the San Raffaele Scientific Institute Internal Review Board (TIGET06). Written informed consent was obtained from parents and/or legal guardians for sample collection. A description of all HDs and patients is reported in Supplemental Tables 1, 2, respectively. In our study patients have been divided in different age groups, which were determined based on previous reports (21) (Supplemental Table 3).

### Thymic Tissue Processing: Thymocytes Recovery and TEC Isolation

Thymi were collected during cardiac surgery and maintained in sodium chloride 0.9% physiological solution (S.A.L.F. SpA, Italy) at 4°C until processed. Thymic tissue was cleaned from blood clots and surrounding fat and connective tissue prior processing. About 1 gram of tissue was then collected and fixed in formalin for histological studies. Thymic tissue was cut in small pieces and thymocytes were recovered by mashing with a sterile syringe plunger. Thymocytes were kept on ice in PBS (CORNING, Corning, NY, USA) containing 1% of penicillin/streptomycin (P/S) (ThermoFisher scientific, Waltham, Massachusetts, USA) and 10% FBS (Sigma-Aldrich, Saint Louis, Missouri, USA) to preserve their viability. TEC isolation was performed following an already published protocol (20) and further optimized in our group. Thymic tissue was digested at 37°C with a solution containing Liberase (Roche, Basel, Switzerland) and DNAse I (Sigma-Aldrich, Saint Louis, Missouri, USA) in three cycles of 40, 40, and 30 min, respectively. After each digestion cycle, supernatant was collected and kept at 37°C, upon addition of an equal volume of RPMI (CORNING, Corning, NY) containing containing 10% FBS and 1% P/S. At the end of the digestion, all three fractions were pooled and centrifuged at 1,500 rpm for 5 min. Thymic single cell suspensions were then incubated with anti-human CD45 microbeads (Miltenyi Biotec, Bergisch Gladbach, Germany) and isolated with the autoMACS Pro Separator (Miltenyi Biotec). The CD45-negative fraction was retrieved and tested by multicolor FACS analyses for the expression of thymic epithelial markers.

### Flow Cytometric Analyses

Immunophenotype of thymocytes was performed by flow cytometry multi-color stainings. For the early developmental stages, an eight-color staining was performed on freshly isolated cells using the following mAbs: CD8 VioBlue (clone BW135/80), CD45 APC (5B1), CD7 APC-Vio770 (CD7-6B7), CD1a FITC (REA736), CD34 PE (AC136), CD5 PE-Vio770 (UCHT2), CD3 VioGreen (REA613) and CD4 PerCP (VIT4) (all mAbs are from Miltenyi Biotec).

For the late stages of T-cell differentiation, a seven-color staining was performed on fresh cells isolated from the thymus using the following mAbs: CD1a FITC (clone Rea736), CD8 PE (BW135/80), CD3 APC (BW264/56), CD4 PE-Vio770 (M-T321), CD69 APC-Vio770 (FN50), CD27 VioBlue (M-T271), CD45 PerCP-Vio700 (5B1) (all mAbs are from Miltenyi Biotec).

Thymic Tregs were studied with a seven-color staining performed on freshly isolated thymocytes using the following mAbs: CD8 PE (clone BW135/80), CD4 PE-Vio770 (M-T321), CD31 APC-Vio770 (AC128), CD45 PerCP-Vio700 (5B1) (all from Miltenyi Biotec) and CD25 APC (M-A251) (BioLegend, San Diego, California, USA). The surface staining was followed by the intracellular staining for FoxP3 Alexa Fluor 488 (clone 259D) and Helios Pacific Blue (22F6) (BioLegend) using the FoxP3 Staining Buffer Set (eBioscience, San Diego, California, USA) and following the protocol recommended by the manufacturer.

The RTEs were analyzed with a six-color staining on freshly isolated thymocytes using the following mAbs: CD4 PE-Vio770 (clone M-T321), CD8 PE (BW135/80), CD45 APC (5B1), CD95 APC-Vio770 (DX2) (all from Miltenyi Biotec), CD45RA PerCP/Cy5.5 (HI100) and CD31 FITC (WM59) (BioLegend).

Peripheral blood mononuclear cells (PBMCs) were isolated by density gradient centrifugation using Lymphoprep™ (density: 1.077 g/ml; STEMCELL Technologies, Vancouver, Canada). To discriminate naïve and memory T cell subsets (central memory, effector memory and TEMRA) we performed an eight-color staining using the following mAbs: CD45 APC (clone 5B1), CD3 VioGreen (REA613), CD4 PE-Vio770 (M-T321), CD8 PE (BW135/80), CD27 VioBlue (M-T271), CD197 (CCR7) FITC (REA546), CD31 APC-Vio770 (AC128) (all from Miltenyi Biotec), and CD45RA PerCP/Cy5.5 (HI100) (BioLegend). To identify the RTE subset, PBMCs were stained with the following mAbs: CD45 APC (clone 5B1), CD3 VioGreen (REA613), CD4 PE-Vio770 (M-T321), CD8 PE (BW135/80), CD27 VioBlue (M-T271), CD31 APC-Vio770 (AC128) (all from Miltenyi Biotec), CD45RA PerCP/Cy5.5 (HI100) (BioLegend).

Peripheral Tregs were analyzed using the following mAbs: CD4 PE-Vio770 (clone M-T321), CD45 APC-Vio770 (5B1) (all from Miltenyi Biotec), CD25 APC (M-A251), CD45RA PerCP/Cy5.5 (HI100), CD127 PE (A019D5) (BioLegend). The surface staining was followed by the intracellular staining using FoxP3 Alexa Fluor 488 (259D) and Helios Pacific Blue (22F6) (BioLegend) antibodies after fixation and permeabilization (FoxP3 Staining Buffer Set, eBioscience), following the protocol recommended by the manufacturer.

TECs were characterized using the following mAbs: CD326 (EpCAM) PE-Vio770 (clone HEA-125), CD45 APC, HLA DR PE (all from Miltenyi Biotech), Ulex FITC (Vector Laboratories, Burlingame, California, USA).

Cells were acquired using a FACS CantoII (BD Biosciences, San Jose, CA, USA) and analyzed with Flow Jo Software (FLOWJO, LLC, Ashland, OR, USA).

### RNA Extraction and Gene Expression Analysis

RNA was extracted from TECs after digestion and CD45 cell-depletion using the RNeasy Micro kit (QIAGEN, Hilden, Germany). Reverse transcription of mRNA was performed with the High Capacity Reverse Trancription Kit (Applied Biosystems, Foster City, CA, USA). Real-time PCR was performed using TaqMan Gene expression Assays (Applied Biosystems) and the EagleTaq Universal Master Mix (Roche, Basel, Switzerland). PCR reactions were performed in MicroAmp®Optical 96-well reaction plates (Applied Biosystems) in a final volume of 25 μl and run on the Viia-7 Real-Time PCR machine (Applied Biosystems). Relative quantification of genes was performed with the 2−ΔΔCt method and expressed as fold change relative to the expression of the endogenous control, RPLPO.

### *In vitro* Treg Suppression Assay

Thymocytes were recovered and kept in PBS with 10% FBS to preserve their viability. Complement-mediated lysis was used to deplete CD8^+^ T cells by mixing thymocytes with 1 μg/mL mouse anti-human CD8a mAb (clone OKT-8, eBioscience) and HLA-DR Rabbit Complement (dilution: 1:30, Life Technologies, Carlsbad, CA, USA) at a concentration of 2 × 10^7^ cells/mL for 1 h at 37°C (22). CD8-depleted cells were filtered, washed, and resuspended in 95 μL MACS buffer (Miltenyi Biotec). 5 μL of CD25 Micro-Beads-II/10^7^ cells (Miltenyi Biotec) were then added to the cell suspension and incubated for 15 min at 4°C. Cell suspension was then enriched to high purity for CD25^+^ cells using the autoMACS Pro Separator (Miltenyi Biotec), according to the manufacturer's instructions.

Peripheral Treg cells were isolated using the MACSxpress Treg Isolation Kit (Miltenyi Biotec), following manufacturer's instructions.

Treg suppression assay was performed as an allogeneic assay using HD T conventional (Tconv) cells. CD4^+^ CD25^−^ Tconv cells were labeled with Celltrace Violet (CellTrace™ Violet Cell Proliferation Kit, ThermoFisher SCIENTIFIC, Walthman, Massachusetts, USA) following the manufacturer's instructions. Tconv were stimulated with MACSiBead™ microbeads preloaded with biotinylated anti-CD2, CD3, and CD28 antibodies (Treg Suppression Inspector, Miltenyi Biotec) at a 1:2 ratio (cells:beads). Tregs were added at a Tconv:Treg cell ratio ranging from 1:1 to 1:0.125. After 6 days, cells were recovered and stained using the following mAbs: CD4 PerCP (clone VIT4), CD8 PE (BW135/80), CD45 APC (5B1), CD3 FITC (BW264/56) (all from Miltenyi Biotec). Treg suppression capacity was assessed by evaluating the proportion of proliferating cells, determined as the frequency of cells diluting the Celltrace Violet dye. Cells were acquired using a FACS CantoII (BD Biosciences) and then analyzed with Flow Jo Software (FLOWJO, LLC).

### TREC Quantification

DNA was purified from PBMCs and thymocytes using the QIAamp DNA Blood Mini Kit according the manufacturer's instructions (QIAGEN). The quantification of TRECs was performed by real-time PCR (Viia-7 Real-Time PCR System; Applied Biosystems) using sjTREC forward primer (5′-CAC ATC CCT TTC AAC CAT GCT-3′), reverse primer (5′-TGC AGG TGC CTA TGC ATC A-3′) and probe (5′-FAM-ACA CCT CTG GTT TTT GTA AAG GTG CCC ACT TAMRA-3′). For the housekeeping gene T-cell receptor alpha constant gene (TCRAC) forward primer (5′-TGG CCT AAC CCT GAT CCT CTT-3′), reverse primer (5′-GGA TTT AGA GTC TCT CAG CTG GTA CAC-3′), and probe (5′-FAM-TCC CAC AGA TAT CCA GAA CCC TGA CCCTAMRA-3′) were used. PCR reactions were developed in MicroAmp®Optical 96-well reaction plates (Applied Biosystems) in a final volume of 25 μl. TREC and TCRAC copy number was determined by extrapolating the values from a standard curve, which was obtained by amplifying serial dilutions of a triple-insert plasmid, containing fragments of TRECs, K-deleting excision circles (KRECs), and TCRAC (23). Assessment of TCRAC served as a control for the quality and quantity of genomic DNA in the sample. The mean quantity of TCRAC was divided by two, considering the presence of two TCRAC gene copies per cell. The number of TRECs per 10^6^ PBMCs was calculated with the following formula: [(mean quantity of TRECs/(mean quantity of TCRAC/2)] × 10^6^.

### Histology and Morphometric Analysis

Human tissue samples were formalin-fixed and paraffin-embedded. Sections (1.5 μm) were used for routine haematoxylin and eosin (H&E) staining. The following primary antibodies were used: rabbit anti-CD3 (ThermoFisher Scientific) (1:100; antigen retrieval treatment (art): micro waves in EDTA buffer pH 8.0; incubation (inc): 1 h at RT), mouse anti-CD4 (Biocare Medical, Pacheco, CA, USA) (1:200, art: pressure chamber in DIVA Decloaker 1x (Biocare Medical); inc: 1 h at RT), mouse anti-CD8 (Biocare Medical) (1:150; art: pressure chamber in DIVA Decloaker 1x inc: 1 h at RT), mouse anti-Terminal Deoxynucleotidyl Transferase (TdT) (Leica Biosystem, Wetzlar, Germany) (1: 200; art: thermostatic bath in EDTA buffer pH 8.0; inc: overnight at 4°C), rat anti-human FoxP3 (eBioscience) (1:100; art: thermostatic bath in EDTA buffer pH 8.0; inc: 1 h at RT), rabbit anti human Involucrin (Abcam, Cambridge, UK) (1:100; art: micro waves in EDTA buffer pH 8.0; inc: 1 h at RT), mouse anti-human AIRE (kindly provided by Prof P. Peterson, University of Tartu, Tartu, Estonia) (1:3,000; art: thermostatic bath in EDTA buffer pH 8.0; inc: 1 h at RT). Depending on the primary antibodies used, sections were incubated with Rat-on-Mouse HRP-Polymer (Biocare Medical) or MACH 1™ Universal HRP Polymer Kit (Biocare Medical), and reactions were developed in Biocare's Betazoid DAB and nuclei counterstained with haematoxylin.

Digital images were acquired by an Olympus XC50 camera mounted on a BX51 microscope (Olympus, Tokyo, Japan) with CellF Imaging software (Soft Imaging System GmbH, Münster, Germany). Morphometric analysis was performed using Olympus Slide Scanner VS120-L100 (Olympus, Tokyo, Japan) to acquire digital images and Image-pro software (Olympus) to analyze them.

### Statistical Analysis

All results are expressed as the mean ± SEM if not stated otherwise. Comparisons between proportions were calculated by using the chi-square test (χ^2^ test) (with continuity correction) as stated in the Figure legends. To assess significance, we used one-way ANOVA with Bonferroni post-correction or two-way ANOVA analysis of variance when specified. We also used two-tailed Mann-Whitney test where specified. *p*-values < 0.05 were considered significant. Analysis of cell populations was performed with the non-parametric combination (NPC) test (24).

## Results

### Altered Representation of Thymic Cortex and Medulla in DGS and DS Patients

**Figure 1:**
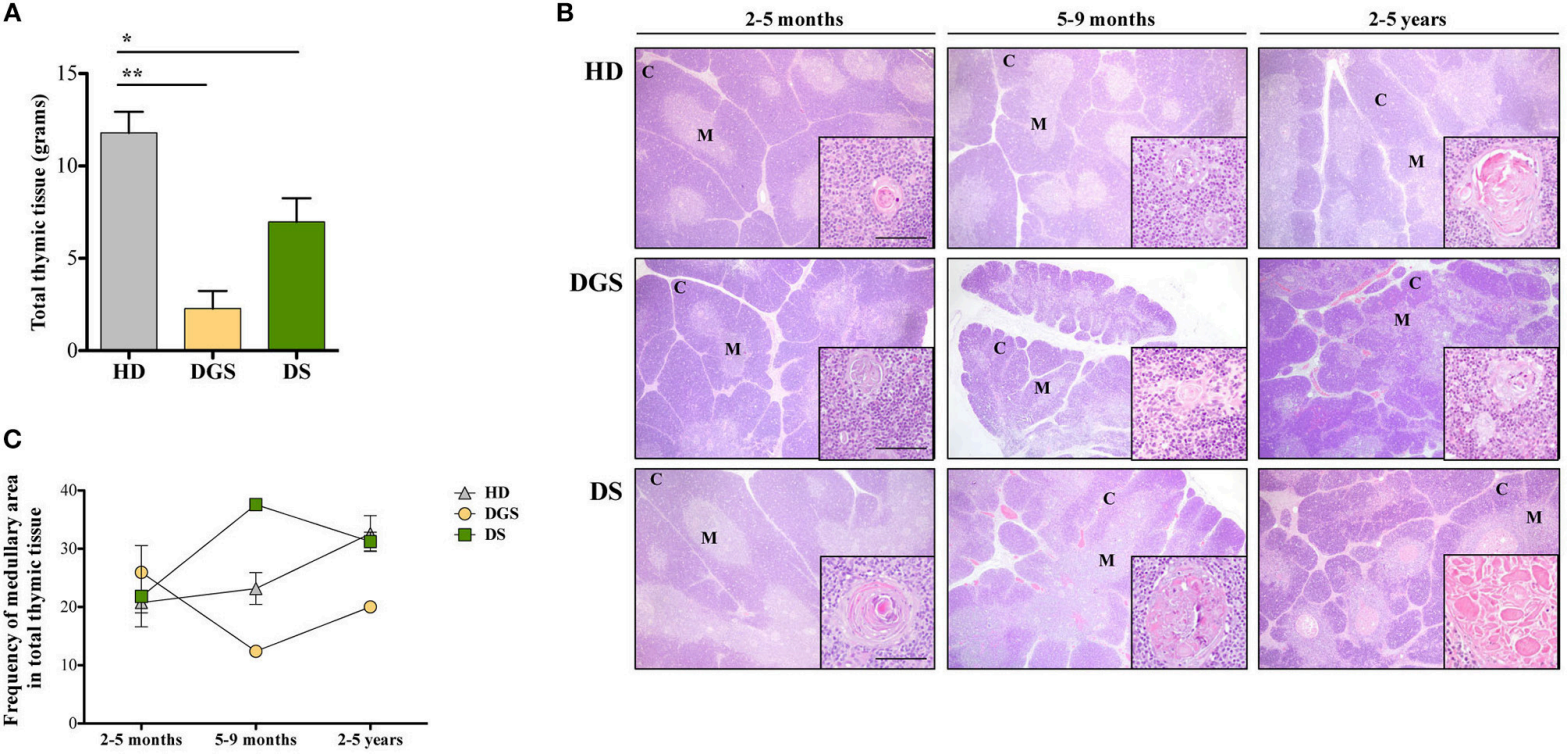
Analyses of thymic size and architecture. **(A)** Thymic size, in terms of grams, in patients as compared to HDs. Mean ± SEM are represented (HD, *n* = 26; DGS, *n* = 5; DS, *n* = 8) (Mann-Whitney test, ^*^p-value < 0.05; ^**^p-value < 0.002); **(B)** Representative H&E staining of thymic cortex (C) and medulla (M) of HDs, DGS and DS patients belonging to the 2–5 months, 5–9 months, and 2–5 years age-groups. Scale bar = 500 μm (main figure) and 100 μm (inset); **(C)** Graphic representation of medullary area evaluated for our patients and compared to HDs (2–5 months: HD, *n* = 3, samples 9, 10, 13 in Supplemental Table 1; DGS, *n* = 1, patient 10 in Supplemental Table 2; DS, *n* = 4, patients 15, 16, 18, 20 in Supplemental Table 2. 5–9 months: HD, *n* = 3, samples 6, 17, 20 in Supplemental Table 1; DGS, *n* = 1, patient 9 in Supplemental Table 2; DS, *n* = 1, patient 17 in Supplemental Table 2. 2–5 years: HD, *n* = 3, samples 21, 23, 24 in Supplemental Table 1; DGS, *n* = 1, patient 8 in Supplemental Table 2; DS, *n* = 2, patients 14, 19 in Supplemental Table 2).

**Figure 2:**
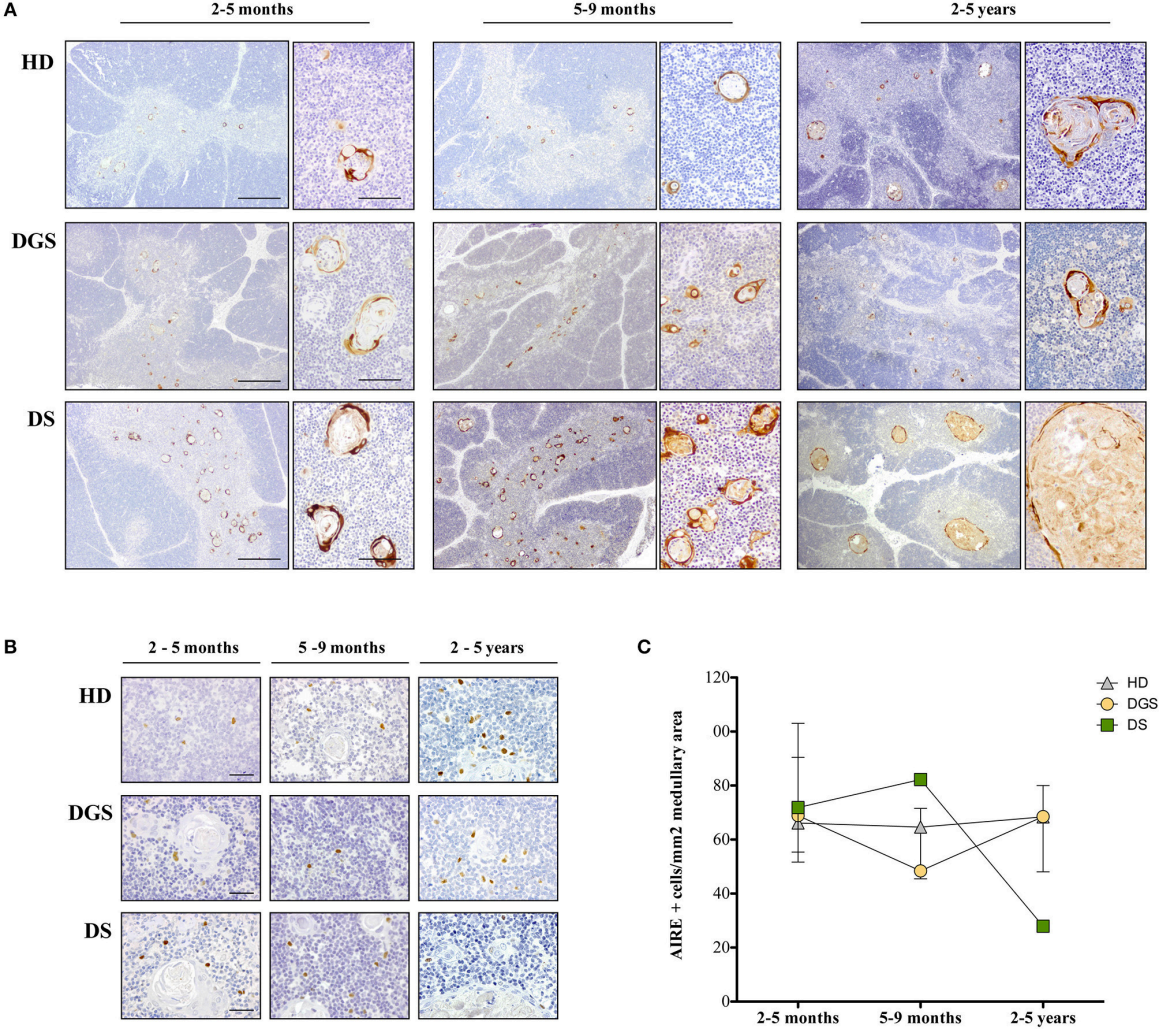
Analysis of thymic maturation and AIRE expression in thymic samples. **(A)** Involucrin immunohistochemical staining in HD, DGS and DS thymic samples. Scale bar = 500 μm (left) and 100 μm (right); **(B)** AIRE immunohistochemical staining in HDs, DGS, and DS thymic samples in different age-groups. Scale bar = 50 μm; **(C)** AIRE^+^ cell counts normalized on medullary area (mm^2^) (2–5 months: HD, *n* = 3, samples 9, 10, 13 in Supplemental Table 1; DGS, *n* = 1, patient 10 in Supplemental Table 2; DS, *n* = 4, patients 15, 16, 18, 20 in Supplemental Table 2. 5–9 months: HD, *n* = 3, samples 6, 17, 20 in Supplemental Table 1; DGS, *n* = 1, patient 9 in Supplemental Table 2; DS, *n* = 1, patient 17 in Supplemental Table 2. 2–5 years: HD, *n* = 3, samples 21, 23, 24 in Supplemental Table 1; DGS, *n* = 1, patient 8 in Supplemental Table 2; DS, *n* = 2, patients 14, 19 in Supplemental Table 2).

In our study, where possible, DGS and DS patients were divided into three different age groups, according to criteria described in literature (21). All thymic samples were weighed in order to evaluate whether the different diseases could affect thymic size. Thymic weight was normalized based on the description of the thymectomy (i.e., total or partial thymectomy). Significant weight reduction was observed in both DGS and DS thymi as compared to age-matched HDs (Figure 1A), irrespective of age-group comparison. We then evaluated the thymic architecture by H&E staining (Figure 1B). The thymic lobes are organized in cortical and medullary areas and the amount of medulla in each lobe reflects the maturation state of the thymic tissue. In fact, immature thymi have majority of their lobes occupied by cortical areas, while, as maturation progresses, there is an increase in medullary areas (25), as shown in the HD (Figure 1B, top panels) and confirmed after quantification in all patients analyzed (Figure 1C). In HDs, the increase in medullary compartment is evident between 5–9 months and 2–5 years age groups (Figures 1B,C). On the contrary, thymi obtained from DGS patients showed no increase in medullary compartment among the different age-groups, indicating a decreased thymic maturation (Figure 1B, middle panels, and Figure 1C). Conversely, DS thymi displayed accelerated maturation kinetics when compared to age-matched HDs, with a peak of thymic maturation in the 5–9 months age-group, as indicated by an increased representation of the medullary area (Figure 1B, lower panels, and Figure 1C). Of note, DS medullary compartment was characterized by the presence of cystic involutions, as shown by their positivity to involucrin staining, a marker expressed by medullary TECs (mTECs) during their last developmental stages and after AIRE downregulation (26) (Figure 2A). Medullary cystic areas can be observed also in thymus from HDs, however they are decreased in number and smaller than those observed in age-matched DS patients. In contrast, DGS patients present very few and small areas of positivity to involucrin (Figure 2A). Altogether, our data indicate the DGS thymic epithelial compartment shows an immature profile, while thymic tissue from DS patients shows an accelerated maturation of the thymic epithelial compartment, with signs of premature involution.

### Altered Kinetics of AIRE Expression in DGS and DS Patients

To further characterize mTEC maturation and function in DGS and DS patients, we evaluated AIRE expression by immunohistochemistry in DGS and DS patients and age-matched HDs (Figures 2B,C). AIRE^+^ cells were detected in all samples analyzed (Figure 2B). Quantification of AIRE^+^ cells, normalized for the total thymic area, in HD thymi in the different age-groups showed that the total number of AIRE^+^ cells remained stable over time with a tendency to increase in the oldest age-group (2–5 years), even in the presence of a high variability in this group (Figure 2C). Conversely, in DGS patients, AIRE^+^ cells progressively decreased showing a drop in the 5–9 months age-group and presented overall numbers lower than those found in HDs (Figure 2C). Remarkably, a dramatic increase in AIRE^+^ cells was observed in thymi obtained from DS patients between the 2–5 months and 5–9 months age-groups, followed by a drastic reduction in the third age-group (2–5 years) (Figure 2C). Higher AIRE expression in thymi from DS patients in the 5–9 months age-group, as compared to age-matched HD samples, was confirmed by analysis of mRNA obtained from digested thymic tissue after depletion of CD45^+^ cells (Supplemental Figure 1). Indeed, we detected a statistically significant increase in expression of AIRE and Ins2, a tissue restricted antigen regulated by AIRE, in DS thymic tissue. The drastic decrease in AIRE expression in the 2–5 years age-group of DS patients well-correlates with the presence of large cystic involutions that can be interpreted as sign of premature aging and decreased thymopoietic activity.

### Thymocyte Development Is Affected in DGS and DS Patients

**Figure 3:**
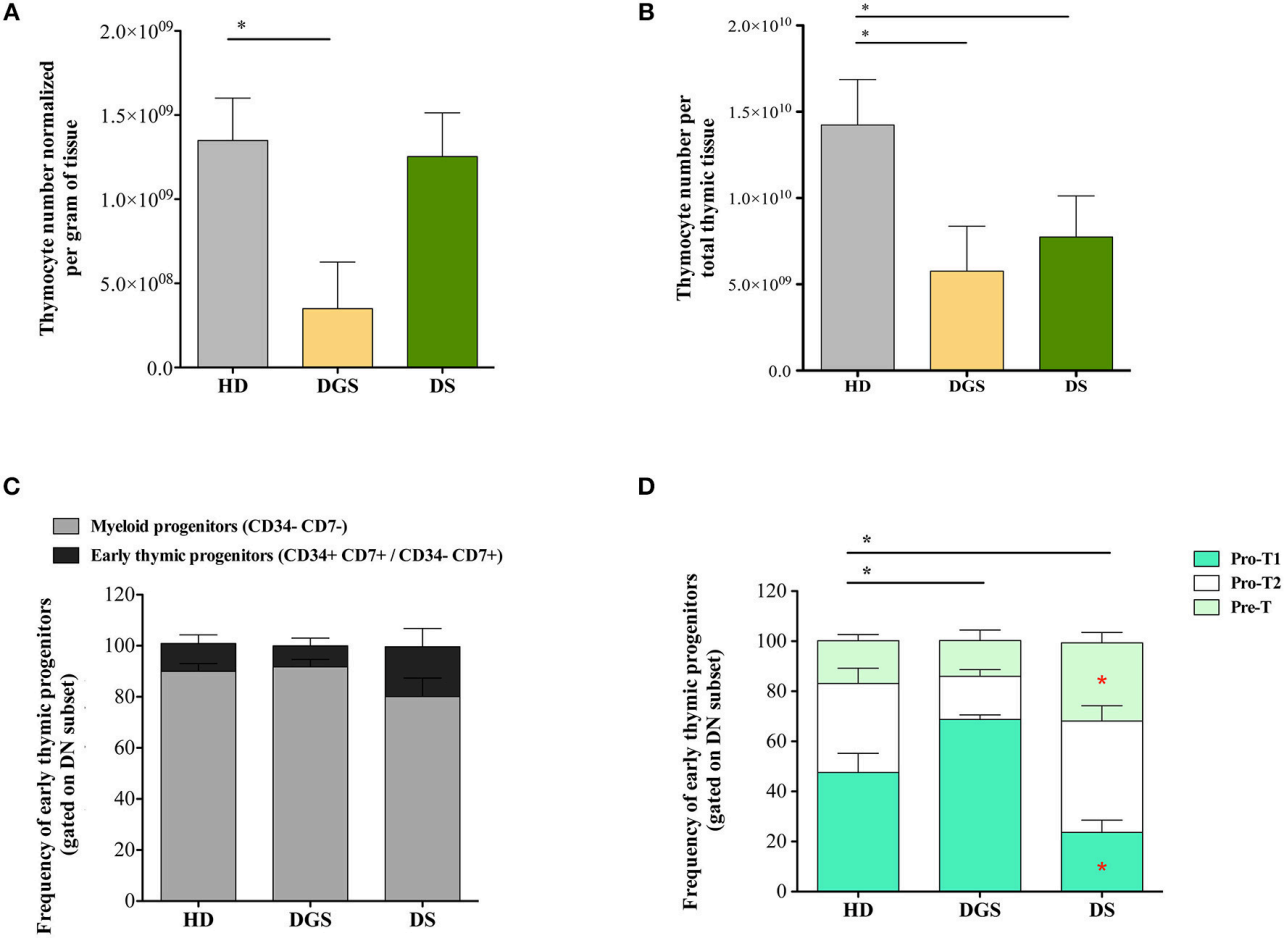
Thymocyte absolute number and analysis of early thymocyte development. **(A,B)** Graphic representation of thymocyte absolute numbers normalized per gram of tissue **(A)** and per total thymus weight **(B)** (HD, *n* = 26; DGS, *n* = 4; DS, *n* = 8) (Mann-Whitney test; ^*^p-value < 0.01. **(C)** Frequencies of Myeloid Progenitors (MP) (CD34^−^ CD7^−^) and early Thymic Progenitors (ETP) (CD34^+^ CD7^+^ and CD34^−^ CD7^+^) calculated on the DN gate (HD, *n* = 26; DGS, *n* = 4; DS, *n* = 8); **(D)** Frequencies of PRO-T1 (CD7^+^ CD5^−^), PRO-T2 (CD7^+^ CD5^+^) and PRE-T (CD1a^+^ CD7^+^ CD5^+^) calculated on the CD34^−^ CD7^+^ gate (HD, *n* = 26; DGS, *n* = 4; DS, *n* = 8). Bars represent medians ± SEM [NPC test: ^*^T-cell subset global distribution, *T-cell subset partial test (HD and DS); ^*^ and *p-value < 0.05].

To evaluate whether alterations in thymic epithelial compartment of DGS and DS patients could affect their thymopoietic function, we analyzed thymocyte generation and differentiation in affected individuals and age-matched HDs. Absolute count of total thymocytes was normalized for gram of tissue collected and for the total amount of thymic tissue. DGS patients showed a significant reduction in the absolute number of thymocytes per gram of tissue, as compared to age-matched HDs, while there was no difference between DS patients and HDs (Figure 3A). However, both DGS and DS patients had reduced absolute counts of thymocytes when the numbers were normalized for the total amount of thymic tissue (Figure 3B). Next, we analyzed in detail thymocyte developmental stages according to the expression of specific markers, as previously reported (27, 28) (Supplemental Figure 2). Myeloid progenitor (MP) and early thymic progenitor (ETP) frequencies were similar in both groups of patients as compared to age-matched HDs (Figure 3C). Next, we characterized the distribution of the subsequent double negative (DN) developmental stages (Figure 3D). Interestingly, we observed a perturbed distribution of PRO-T1, PRO-T2, and PRE-T subsets in all patients. In particular, an increased frequency of PRO-T1 and a reduction of PRO-T2 were present in DGS patients. In DS patients a significant reduction of PRO-T1 cells was observed, while PRO-T2 frequency was significantly increased. Also PRE-T subset was increased in DS patients, as compared to HDs, but this increase did not reach statistical significance. These results indicate a partial block in DN cell development in DGS patients, with an accumulation at the earliest stage, while we could observe an acceleration toward the more mature DN cell subsets in DS patients.

**Figure 4:**
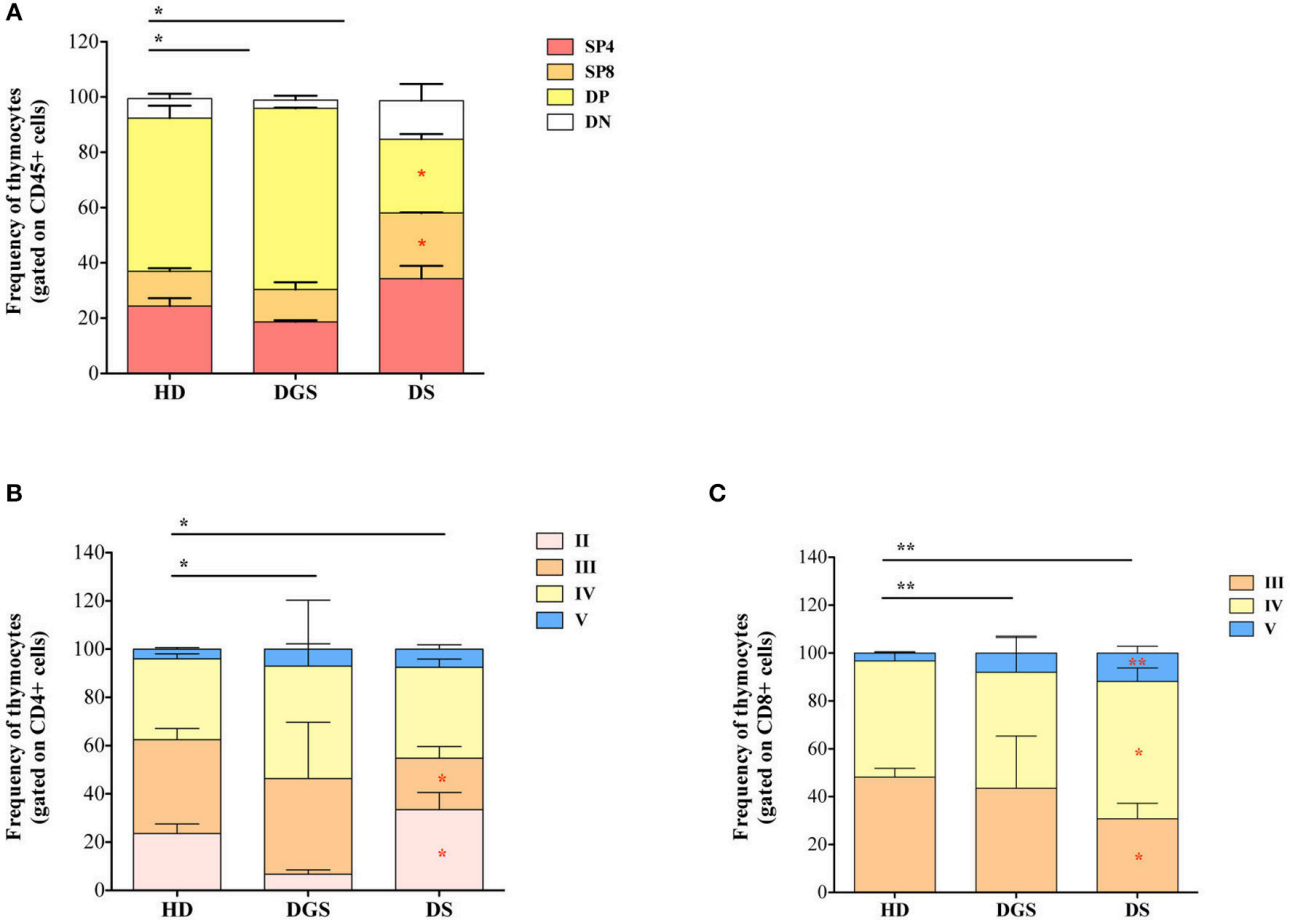
Thymocyte composition and late stages of thymocyte development. **(A)** Frequencies of DN, DP, SP4, and SP8 composing the thymus (HD, *n* = 26; DGS, *n* = 4; DS, *n* = 8); **(B,C)** Frequencies of latest stages of thymocyte development for SP4 **(B)** and SP8 **(C)** with the dissection of their developmental stages starting from the Ist stage to the Vth (HD, *n* = 26; DGS, *n* = 4; DS, *n* = 8); Bars represent medians ± SEM [NPC test: ^*^T-cell subset global distribution, *T-cell subset partial test (HD and DS); ^*^ and *p-value < 0.05; ^**^ and **p-value < 0.002].

We thus examined the later stages of thymic T cell development starting from double positive (DP) stage (Figure 4A). T-cell subset distribution was significantly altered in DGS patients, showing in particular an increase in DP subset, as compared to HDs, in accordance with their more immature thymic profile. Also in DS patients thymocyte distribution was significantly altered as compared to HDs and characterized by a significantly increased percentage of single positive (SP) thymocytes, in particular CD4 single positive thymocytes (SP4), and a significantly reduced proportion of DP cells. When we analyzed more in detail the different stages of SP cell development, we noticed that also these stages were altered in our cohorts of patients as compared to age-matched HDs. SP4 and CD8 single positive thymocytes (SP8) maturational stages were altered in both DGS and DS patients (Figures 4B,C). These results indicate that thymocyte development is mainly affected at the earliest stages in DGS patients, while it is significantly altered in later stages in DS patients, in whom there is a clear skewing toward increased thymocyte maturation.

### Reduced Absolute Counts and Function of Thymic and Peripheral Regulatory T Cells in DGS and DS Patients

**Figure 5:**
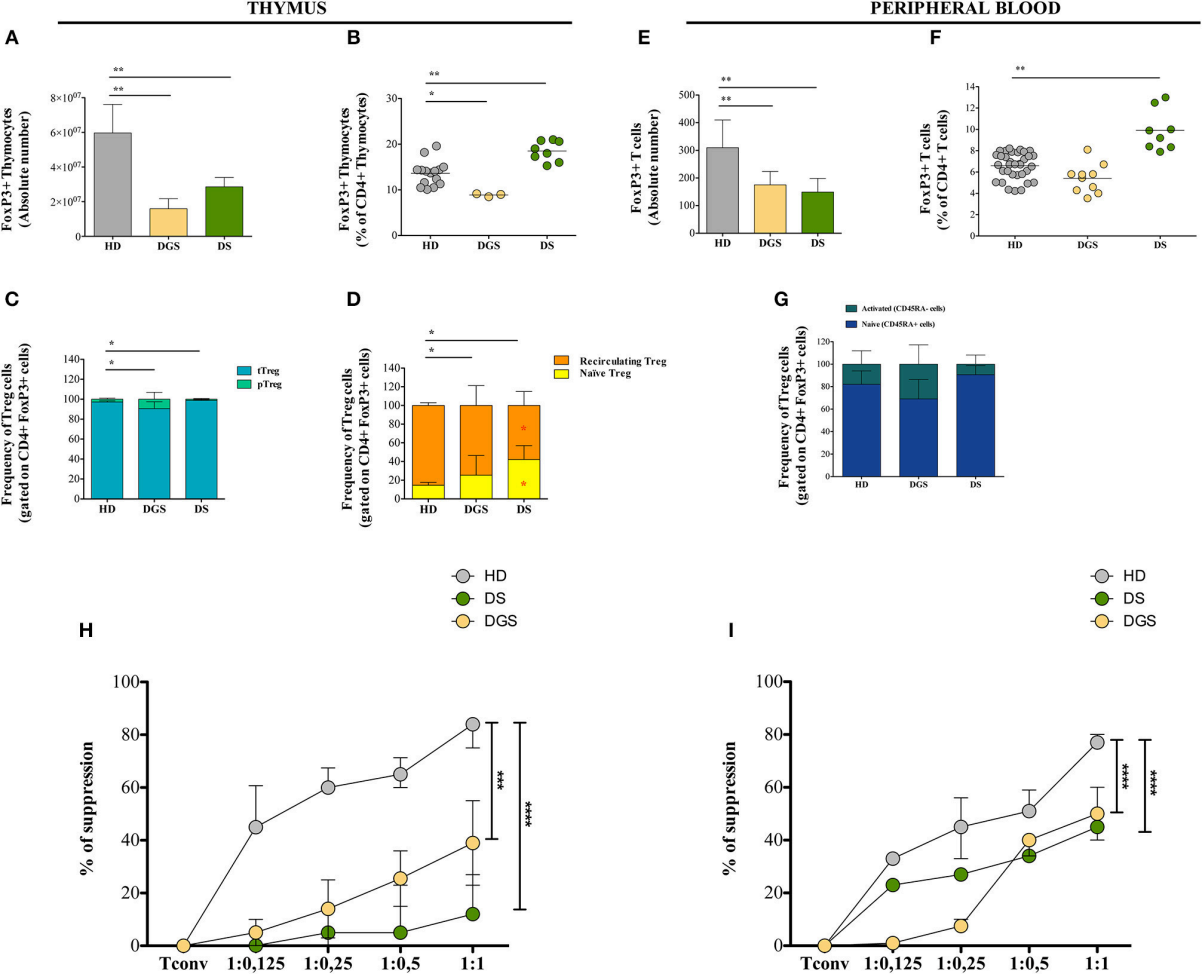
Thymic and peripheral Treg cell distribution and analysis of their suppressive function in DGS and DS patients. **(A–D)** Absolute counts (HD, *n* = 15; DGS, *n* = 3; DS, *n* = 8) **(A)**, frequency (HD, *n* = 15; DGS, *n* = 3; DS, *n* = 8) (Mann-Whitney test; ^*^p-value < 0.01; ^**^p-value < 0.002; ^***^p-value < 0.0001) **(B)**, and phenotype **(C,D)** of thymic Treg cells [NPC test: ^*^T-cell subset global distribution, *T-cell subset partial test (HD and DS); ^*^ and *p-value < 0.05] (HD, *n* = 15; DGS, *n* = 3; DS, *n* = 8); **(E–G)** Absolute counts **(E)**, frequency **(F)**, and phenotype **(G)** of peripheral Treg cells (HD, *n* = 34; DGS, *n* = 10; DS, *n* = 8) (Mann-Whitney and NPC test; ^*^p-value < 0.01; ^**^p-value < 0.002; ^***^p-value < 0.0001). Mean ± SEM are represented; **(H,I)** Thymic (HD, *n* = 5; DGS, *n* = 4; DS, *n* = 4) **(H)** and peripheral (HD, *n* = 5; DGS, *n* = 4; DS, *n* = 4) **(I)** Treg suppressive ability tested at different Tconv:Treg ratios (Two-way ANOVA; ^***^p-value < 0.001; ^****^p-value < 0.0001). Mean ± SEM are represented. Suppressive ability of Tregs is calculated with the following formula: [(% of proliferation of Tconv alone – % of proliferation of Tconv and Treg at different ratios)/(% of proliferation on Tconv alone)]^*^100.

Next, we investigated Treg cells in the thymus and in parallel in peripheral blood of DGS and DS patients, evaluating their central and peripheral absolute counts and function. Flow cytometric analysis showed reduced absolute number and frequency of FoxP3^+^ thymocytes in DGS patients (Figures 5A,B). Conversely, DS patients showed an increased frequency of thymic Tregs (Figure 5B) but reduced absolute counts as compared to HDs (Figure 5A). We then analyzed the different subsets of thymic Tregs, including thymic Tregs (Helios^+^), peripheral Tregs (Helios^−^), naïve Tregs (CD31^+^) and recirculating Tregs (CD31^−^) (Figures 5C,D), even if the role of Helios is still subject to debat (29–31). The proportion of thymic Tregs was found to be significantly decreased in DGS patients and increased in DS patients, as compared to HDs (Figure 5C). Additionally, we noticed a significantly increased frequency of naïve Tregs and a consequent reduction in recirculating Tregs in both DS and DGS patients (Figure 5D).

Moving to the peripheral blood, we found decreased absolute counts and frequencies of Tregs in DGS patients (Figures 5E,F), while in DS patients their absolute counts were reduced but they had increased frequency (Figures 5E,F), similarly to what found in the thymus. Peripheral human FoxP3^+^ Tregs can be classified as naïve (defined as CD4^+^CD25^+^FoxP3^+^CD45RA^+^ cells) or memory/activated (defined as CD4^+^CD25^+^FoxP3^+^CD45RA^−^ cells), which derive from active and proliferating CD45RA^+^ Tregs. We noticed a perturbed distribution of these subsets in our patients as compared to age-matched HDs, with an increased frequency of activated Tregs in DGS patients, while DS patients showed an increased frequency of naïve Tregs (Figure 5G).

To assess Treg suppressive activity in DGS and DS patients, Treg suppression assay was performed by testing Tregs obtained from thymus or peripheral blood in an allogenic setting upon incubation with Tconv cells (CD4^+^CD25^−^) stimulated *in vitro* with a pan-T stimulus (anti-CD2, anti-CD3, and anti-CD28-coated beads) (Figures 5H,I). Tregs were added to the culture at different ratios and proliferation of Tconv cells was analyzed after 6 days of culture. Our results showed a reduced suppressive ability of Tregs from all patients analyzed as compared to Tregs isolated from HDs. Of note, the defective Treg suppressive ability in patients was more profound in Treg cells isolated from thymus as compared to peripheral Tregs.

### Reduced Thymic Output in Both DGS and DS Patients

**Figure 6:**
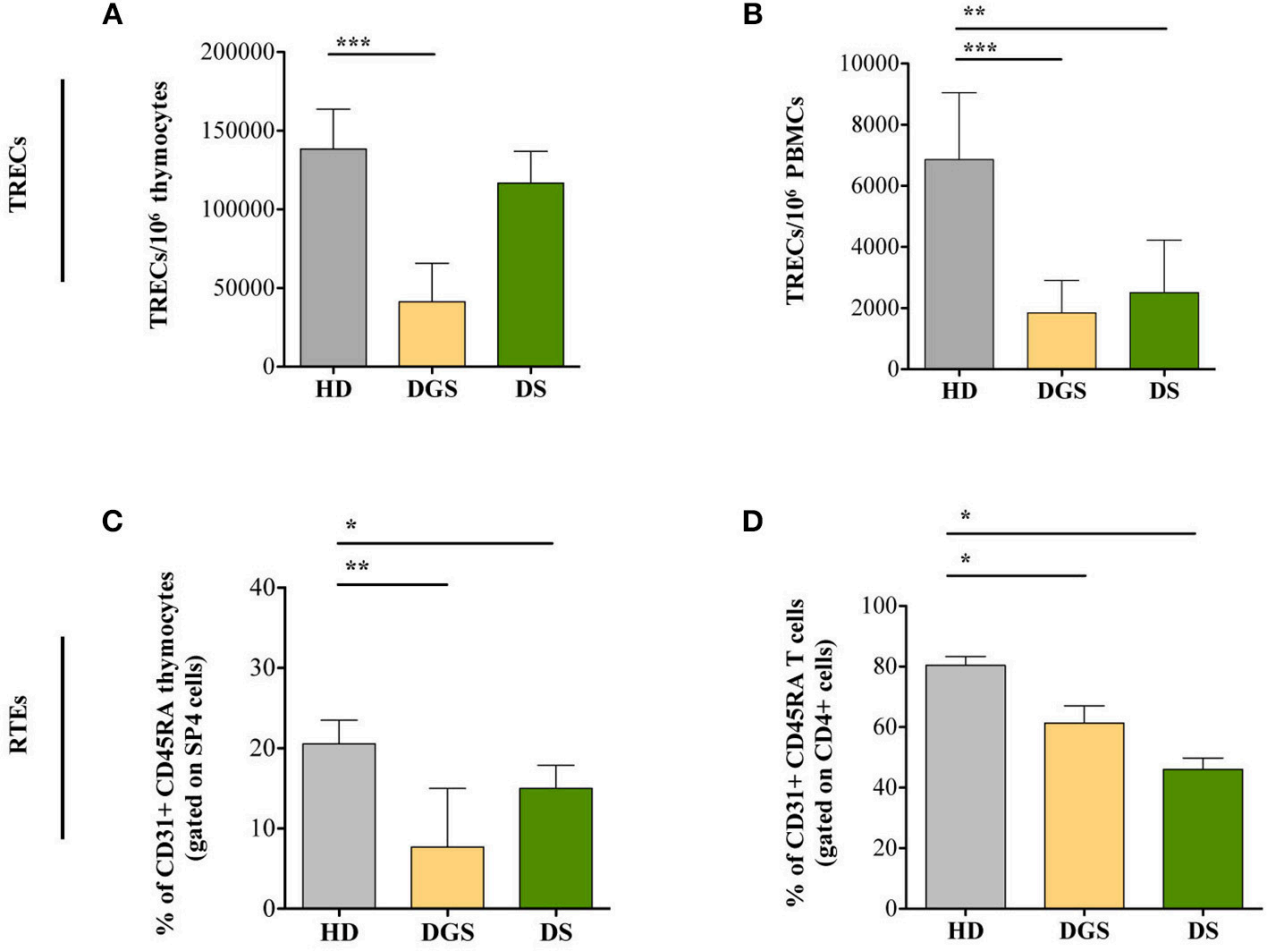
Evaluation of TRECs and RTEs in children with DGS and DS. **(A)** sj-TREC per million of thymocytes (HD, *n* = 26; DGS, *n* = 4; DS, *n* = 8); **(B)** *sj*-TREC per million of PBMCs (HD, *n* = 34; DGS, *n* = 10; DS, *n* = 8); **(C,D)** Frequencies of RTEs among thymocytes (HD, *n* = 26; DGS, *n* = 4; DS, *n* = 8) **(C)** and among PBMCs (HD, *n* = 34; DGS, *n* = 10; DS, *n* = 8) **(D)**. Mean ± SEM are represented (Mann-Whitney test; ^*^p-value < 0.05; ^**^p-value < 0.01; ^***^p-value < 0.0001).

To further characterize thymic function, we quantified T-cell receptor excision circles (TREC) and frequency of recent thymic emigrants (RTEs) both in the thymus and in peripheral blood of DGS and DS patients. The *sj*TREC measurement is a direct quantification of the intrathymic proliferation step occurring between the β- and α-chain rearrangements, providing a measure of thymic function (23). Our results showed a significant reduction in TREC numbers in DGS patients in both thymus and peripheral blood (Figures 6A,B, respectively). Conversely, DS patients showed a drastic reduction of TREC numbers only in the peripheral compartment but not in the thymus (Figures 6A,B). To confirm these data, we evaluated a subset of naïve T lymphocytes (CD4^+^CD45RA^+^CD31^+^), with a low proliferative history, considered to be the most recent thymic emigrants (32). In agreement with a reduced thymic output, frequency and absolute counts of CD31^+^ naïve T lymphocytes were diminished in DGS and DS patients both in the thymus and in periphery, reaching a statistical significance in both compartments (Figures 6C,D and Supplemental Figure 3).

### Decrease in Naive CD4^+^ and CD8^+^ T Cells in DGS and DS Patients

**Figure 7:**
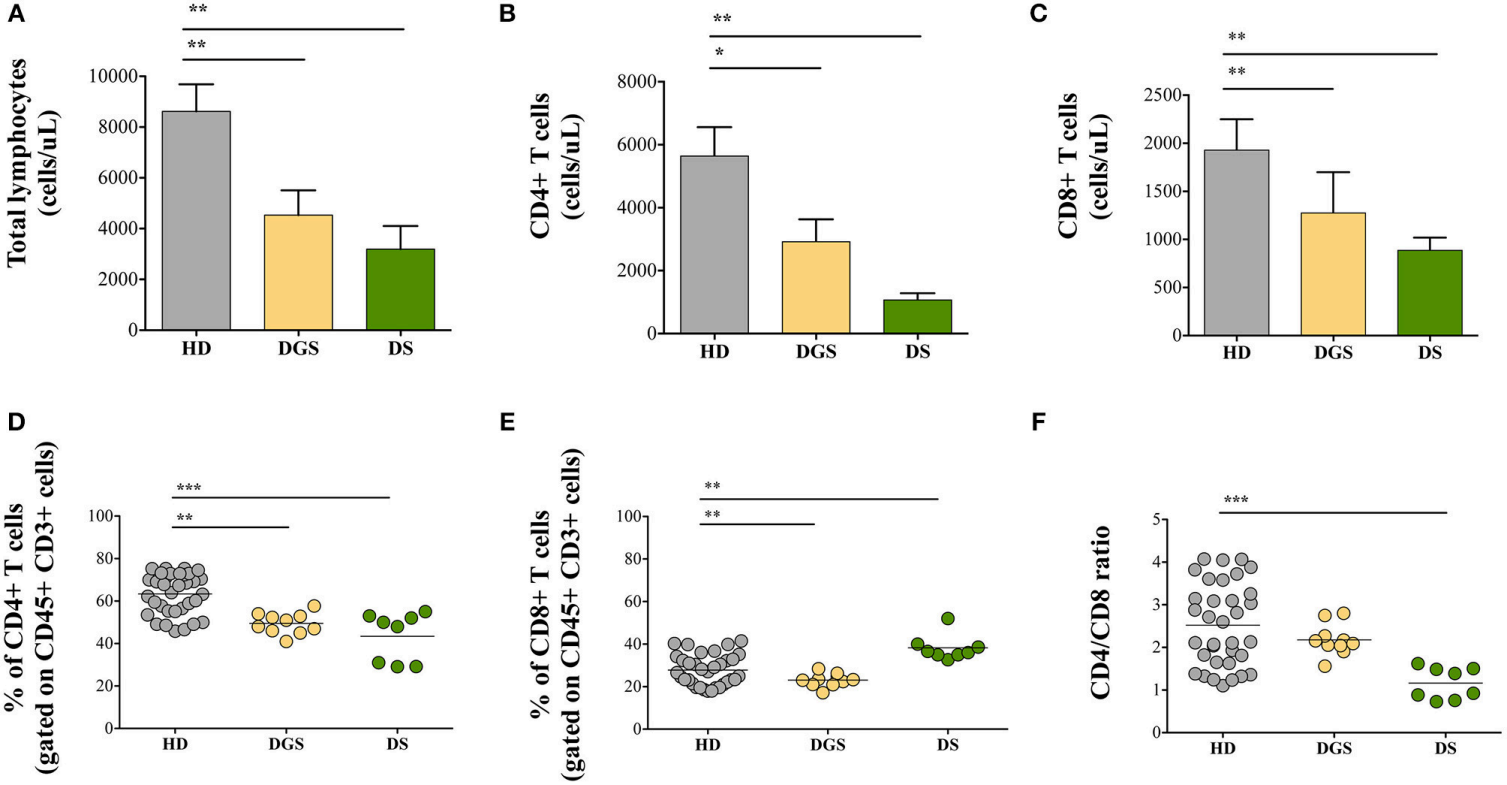
Total lymphocytes and distribution of CD4^+^ and CD8^+^ T-cell subsets. **(A)** Absolute counts per microliter of total blood of lymphocytes (HD, *n* = 34; DGS, *n* = 10; DS, *n* = 8); **(B,D)** Absolute counts and frequencies of CD4^+^ cells (HD, *n* = 34; DGS, *n* = 10; DS, *n* = 8); **(C,E)** Absolute counts and frequencies of CD8^+^ cells (HD, *n* = 34; DGS, *n* = 10; DS, *n* = 8); **(F)** CD4/CD8 T lymphocyte ratios of HD, DGS, and DS patients. Mean ± SEM are represented (Mann-Whitney test; ^*^p-value < 0.01; ^**^p-value < 0.002; ^***^p-value < 0.0001).

**Figure 8:**
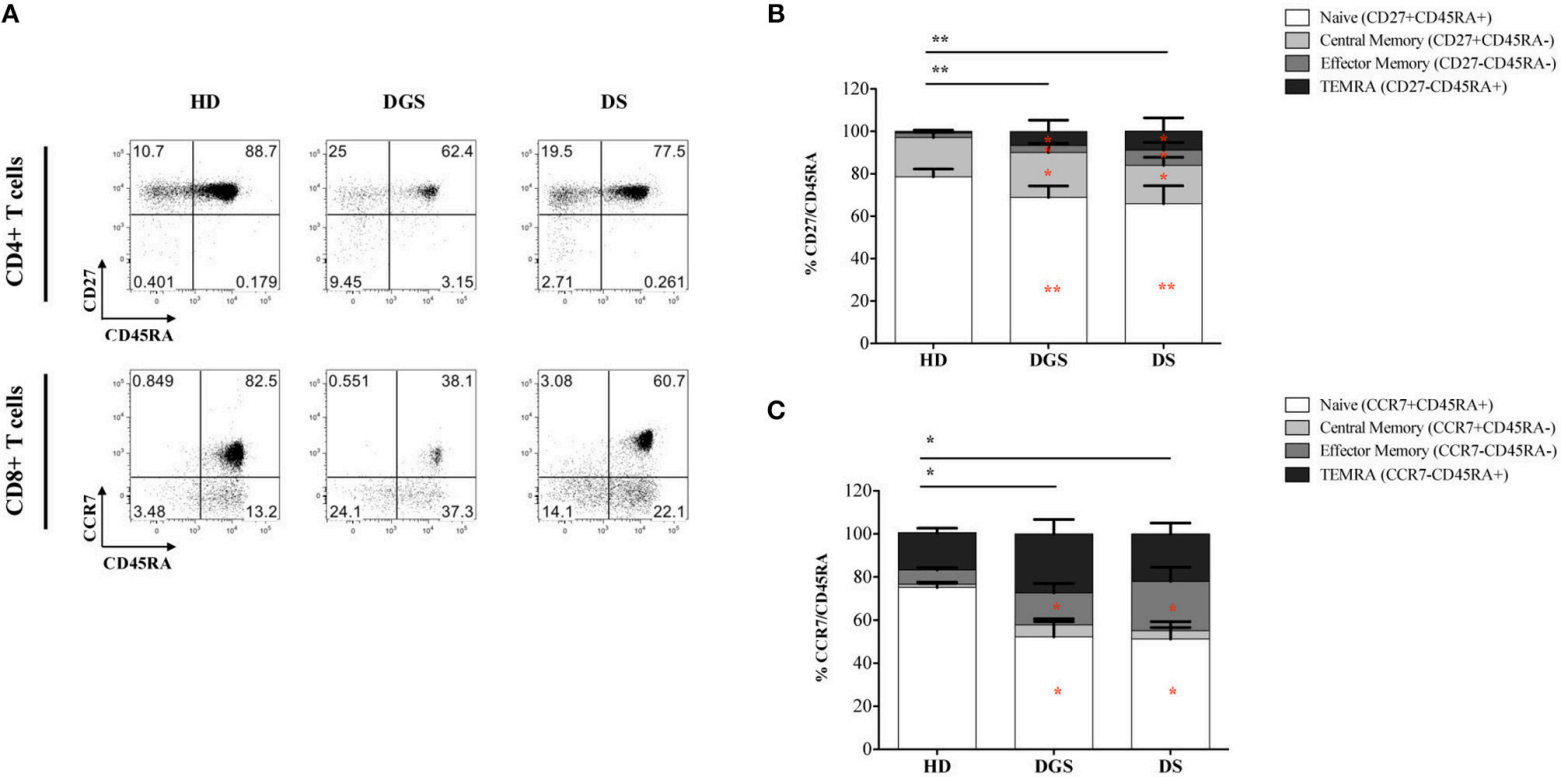
Analyses of peripheral T-cell immunophenotype and gating strategy. **(A)** Gating strategy used to analyze the maturation stages of CD4^+^ and CD8^+^ T cells; **(B,C)** Frequency of CD4^+^ **(B)** and CD8^+^ **(C)** T-cell subsets in all groups of patients and age-matched HDs (HD, *n* = 34; DGS, *n* = 10; DS, *n* = 8). Mean ± SEM are represented [NPC test: ^*^T-cell subset global distribution, *T-cell subset partial test (HD and DS); * and *p-value < 0.05; ^**^ and **p-value < 0.002; ^***^p-value < 0.0001).

In parallel to the analysis of the thymic tissues, we evaluated T cell subset distribution and absolute counts in peripheral blood of DGS and DS patients. Our results show a significant decrease of total lymphocyte counts in both cohorts of patients (Figure 7A). The absolute counts were performed also for CD4^+^ and CD8^+^ T-cell subsets, confirming the reduced numbers in DGS and DS patients in both subsets, as compared to controls (Figures 7B,C). Similarly, the frequency of both CD4^+^ T cells in DGS and DS patients were found to be reduced, as compared to HDs (Figure 7D). CD8^+^ T cell frequency was also reduced in DGS patients, while their proportion in DS patients was found to be slightly increased (Figure 7E). These results were confirmed also by CD4/CD8 T lymphocyte ratios, confirming an imbalance in DS patients (Figure 7F). Finally, analysis of naïve, central memory and effector memory T cell subset frequency (Figure 8A) showed a reduction in naïve CD4^+^ and CD8^+^ T cells, while an increased frequency in the memory compartment in both cell subsets in DGS and DS patients (Figures 8B,C). Altogether, these findings demonstrate that DGS and DS patients have a perturbed peripheral T-cell distribution characterized by the decrease of naïve T cells, confirming their reduced thymic functionality.

## Discussion

To study to which extent primary defects in thymic epithelial compartment can affect establishment of tolerance and result in immune dysregulation, we exploited two paradigmatic human diseases due to chromosomal abnormalities: DGS, caused by the deletion of the long arm of chromosome 22 at p11.2, and DS, characterized by the presence of an extra copy of all or part of chromosome 21. In partial DGS, a primary thymic defect leading to impaired central tolerance was initially suggested because of the presence of autoimmune signs associated with recurrent infections (5). Indeed, a compromised central tolerance, in terms of positive and negative selection of emerging T cells, could lead to the escape of autoreactive thymocytes and/or disturbed Treg cell generation and, consequently, to the development of a dysfunctional immune system. Thymic hypoplasia has been described in the majority of patients affected by DGS (12, 13, 31) and further confirmed in our study, in which we observed thymic size reduction in DGS patients as compared to HDs. These observations were corroborated by the evidence of an abnormal thymic organization, showing an altered distribution of cortex and medullary area, with reduced representation of the medullary compartment in all age-groups of DGS patients. A disorganized architecture, with poor defined cortical and medullary demarcation, is reminiscent of an immature organ. To further characterize the thymic epithelial compartment, we analyzed maturation (Involucrin) and functional (AIRE) markers by immunohistochemistry. Analysis of Involucrin and AIRE which are selectively expressed by mTECs showed a reduction in the expression on both markers, indicating an impairment in thymic epithelium functionality, particularly in the medullary compartment which is involved in the negative selection of autoreactive thymocytes (33). Indeed, AIRE is expressed only by a subset of mature mTECs (34), so it is possible that in DGS mTECs might not reach a fully mature phenotype, resulting in a reduced expression of AIRE and consequently impaired expression of AIRE-dependent tissue restricted antigens. These evidences indicate that the immature status of the DGS thymus might render it unable to properly sustain thymocyte selection because of the defective lympho-epithelial cross talk, as described in other severe combined immunodeficiencies (35, 36). Based on these findings, we can speculate that the severity of the immune deficiency could reflect the gravity of thymic epithelial defect. Thus, it is likely that the degree of thymic tissue immaturity might influence the severity of DGS immunological status, which can range from near normal to completely deficient in patients with a profound thymic hypoplasia. Overall, these findings would support the hypothesis of an impaired central tolerance due to an altered thymic maturation and a reduced AIRE expression leading to the escape of autoreactive T cells. The immature profile of the thymic compartment is reflected also at the earliest stages of thymocyte development. Indeed, our observations showed a reduced number of thymocytes populating DGS thymus as compared to age-matched HDs. Furthermore, analysis of thymocyte development showed perturbed maturation kinetics throughout different maturation steps with an accumulation at DP stage. Thus, cells that further mature in SP thymocytes are very few with respect to HDs. This phenomenon might reflect the incomplete mTEC maturation and their reduced ability to attract thymocytes from the cortical to the medullary area, through chemokine receptors. The reduced number of thymocytes reaching the SP status is reflected also in the reduced number of T cells populating the peripheral compartment. It is known that the maintenance of the peripheral T-cell pool is achieved through both the generation of new thymocytes egressing the thymus to enrich the pool of recirculating T cells and the homeostatic proliferation of peripheral T lymphocytes (35). Thus, diminished thymic output and the consequent lymphopenia could affect the immune system development and compromise the integrity of T-cell immunity during adulthood. In agreement with previous studies (12, 27, 37), we observed a reduced thymic output in DGS patients contributing to the decreased numbers of peripheral T lymphocytes. Indeed, CD4^+^ T lymphocytes circulating in the peripheral blood of DGS patients, showed an increased proliferation history, reflected as a reduced frequency of CD31^+^ cells in the CD4^+^CD45RA naïve T-lymphocyte compartment. Thus, these patients are characterized by an accelerated conversion of naïve to memory T cells both in CD4^+^ and CD8^+^ T cell subsets. The skewing toward a memory phenotype and the progressive exhaustion of naïve T cell pool is favored by the recurrent infections occurring in these patients. In addition, the reduction in the naïve CD4^+^ T cell numbers has been associated with an increased incidence of autoimmunity in patients with DGS (18). As expected, the reduced capacity of DP thymocytes to develop into SP T cells affects also the generation of Tregs that are reduced in terms of absolute numbers with an imbalance in the naïve subset (CD45RA) both in the thymus and peripheral compartment. In peripheral blood, we found an inverted ratio of naïve and activated Tregs as compared to HDs. Treg numbers are correlated to the number of activated CD4^+^ T cells in order to control T-cell expansion during immune responses, thus preventing autoimmune or lymphoproliferative disease (12). Thus, the relative increase of activated CD4^+^ T cells due to their homeostatic expansion, associated with the reduction of naïve T cells and relative increase of the memory compartment, might favor Treg activation leading to the exhaustion of their naïve pool. Consequently, the prevalence of a memory/activated Treg subset could jeopardize their homeostatic role contributing to the increased susceptibility to develop infection and autoimmune signs (12, 13). Consistently, the analysis of Treg functionality clearly demonstrates defective function, which is more evident in Treg thymic compartment. It is reported that in patients with autoimmune polyendocrinopathy-candidiasis-ectodermal dystrophy (APECED), the naïve Treg pool shows an accelerated turnover and a shift toward an activated phenotype that impairs their suppressive function and results in autoimmune phenomena (38). Similarly, we can speculate that besides the thymic defect leading to impaired Treg cell generation, the defect in Treg suppression could contribute to the chronic inflammatory environment, which in turn leads to the exhaustion of Treg cells further impinging their ability in maintaining the immune homeostasis.

In summary, thymic analysis in DGS has allowed us to provide an additional mechanism of immune dysregulation acting from the early phases of thymocyte maturation and caused by an incomplete maturation of the thymic epithelium. Consistently, the thymic output is decreased with a reduction of the T-cell peripheral compartment both in terms of absolute numbers and proportion as compared to age-matched HDs. Thus, DGS patients present a deficit in T cells and an increased homeostatic expansion of both CD4^+^ and CD8^+^ T cell compartments, which might lead to a perturbation of the TCR repertoire and might be associated to an accelerated aging of the immune system. In addition, perturbation in CD4^+^ T-cell subset distribution and reduction in absolute Treg number promote skewing of Treg cells toward a memory/activated phenotype compromising their suppressive function. All these findings indicate severe impairment of the central and peripheral tolerance mechanisms, which in association with antigen overloading could favor autoimmune phenomena in pDGS patients.

Similarly to DGS patients, DS patients are characterized by high susceptibility to develop autoimmune phenomena and recurrent infections (39) and our data provide evidence that also in DS the reduced thymic function is a causative factor supporting both the decreased response to infections and the increased susceptibility to develop organ specific autoimmune disorders. DS thymi show abnormal architecture with an increase of the medullary compartment indicating an accelerated maturation kinetics or premature involution. These observations were also supported by the presence of enlarged cystic formations, likely derived from the massive degeneration of Hassal's bodies, progressively replacing the medulla compartment and affecting its function. Our hypothesis of a senescent thymus is also supported by the abnormal distribution of cortical and medullary compartment in the different age-groups, in particular in older patients. In fact, we could observe that DS patients already between the age-groups of 5–9 months and 2–5 years have a clear decrease in AIRE^+^ cells/area, as compared to the age-matched HD, and we hypothesize that they keep decreasing in time and more quickly than in HD. Our hypothesis is supported by data previously published on DS patients (ranging from 4 months to 12 years) in which AIRE^+^ cell number and thymus functionality were found decreased as compared to age-matched HD (40, 41). Indeed, the premature expansion of the medullary compartment, resulting in an enlargement and a precocious involution of the Hassal's bodies, results in a diminished capacity to drive thymocyte development and provide them a proper education. These findings indicate that DS thymi lose their function early in childhood, anticipating the age-related involution process normally occurring after puberty. In parallel, we observed a reduced frequency of early thymic progenitors and an expansion of PRO-T2 and PRE-T more mature stages, indicating that thymocytes are forced to an accelerated maturation occurring in the cortical area. The reduction of immature thymocytes could also be due to a decreased proliferation and an increased apoptosis of the immature PRO-T1 populations, as shown in mice in DN1 and DN2 stages (17). The altered kinetics in thymocyte maturation impinges the cross-talk between thymocytes and epithelial cells contributing to further impair the establishment of central tolerance (34, 42, 43). The perturbed kinetics of AIRE expression, detected in DS thymi, may result in improper education and consequent egression of autoreactive SP T cells. The alterations found in the DS thymus could also contribute to the impairment of Treg cell compartment. Indeed, Tregs, despite being increased in frequency, are defective in suppressive function (44). It could be possible that the accelerated development characterizing DS thymi could influence Treg cell development compromising their normal functionality. Thus, the higher incidence of autoimmune diseases in DS may also be related, at least in part, to the functional impairment of Treg cells. Altogether our data reinforce the pivotal role of defective central tolerance in the pathogenesis of autoimmune disorders in DS patients.

The thymus in DS individuals is smaller and hypocellular, even in infants, containing a decreased proportion of phenotypically mature thymocytes. The characteristic hypocellularity present in DS thymi is reflected also in the number of TRECs. Indeed, significantly lower numbers of TRECs in peripheral blood cells are found in DS children in comparison with HDs. These results are supported also by lower numbers of peripheral lymphocytes and by an imbalance in their subsets, with a reduction in CD4^+^ and CD8^+^ T cell naïve subsets and a consequently increased frequency of memory cells. Altogether these findings could be interpreted as an early senescence of the immune system in DS patients. Indeed, naïve helper and cytotoxic T lymphocytes as well as TRECs in peripheral blood cells decrease with aging, while central and effector memory T helper lymphocytes, effector memory and terminally differentiated cytotoxic T lymphocytes increase, similarly to what we found already in very young DS patients. Thus, we can conclude that in DS patients the immune system quickly declines with age, with kinetics faster than those taking place in age-matched HDs, leading to an increased susceptibility to infections and a higher incidence of autoimmune phenomena (16, 17, 19). Indeed, DS patients prematurely show clinical manifestations that are normally seen with aging (44, 45). In summary, our study suggests that accelerated maturation of thymic epithelial cell compartment, leading to a premature decrease in thymic functionality may be involved in the establishment of immunological abnormalities in DS patients. These alterations may affect the thymic selection process of T conventional cells and the establishment of the Treg cell pool and determine the increased susceptibility to develop autoimmune diseases typical of DS patients.

Overall, although our results on the thymic epithelium architecture and thymocyte development would need to be further confirmed in larger cohorts of DGS and DS patients, our study provides for the first time evidence of the extent to which thymic epithelium defects, leading to a decrease thymic tissue maturation, as in the case of DGS patients, or to an accelerated maturation and consequent thymic early involution, as in the case of DS patients, can contribute to immune dysregulation sustaining on one hand increased susceptibility to infections and on the other hand predisposing these patients to develop autoimmune manifestations.

## Author Contributions

GEM, AV, and MB designed and performed research, analyzed data, and wrote the paper. IB, FF, and EF contributed to perform experiments and data interpretation. FF and DA contributed to the drafting and management of the clinical protocol. LI provided reagents and contributed to data interpretation. DA, SD, CC, PP, and LN contributed to data interpretation. EC, DA, SB, GM, VD, FC, GF, AC, and AG provided human samples and clinical information. All authors have critically revised and approved the manuscript.

### Conflict of Interest Statement

The authors declare that the research was conducted in the absence of any commercial or financial relationships that could be construed as a potential conflict of interest.

## Supplementary Material

Supplemental Figure 1 Increased AIRE and Ins2 expression in thymic tissue from DS patients. Comparison of mRNA expression of AIRE and tissue restricted antigen AIRE-dependent, Ins2, in HDs and DS patients, normalized for the expression of the housekeeping gene RPLPO (HD, *n* = 3; DS, *n* = 3). Mean ± SEM are represented (Two-way ANOVA test; ^**^*p*-value < 0.002).

Supplemental Figure 2 Thymocyte maturational stages from double negative stage to mature T lymphocytes. Sequential view of the different phenotypical markers expressed during thymocyte differentiation and thymic emigration of CD4^+^ and CD8^+^ T cells. Different maturational stages can be defined by the relative expression of specific markers, such as CD69, CD27, CD7, and CD1a [modified from Vanhecke et al. (27); Reimann et al. (28)].

Supplemental Figure 3 Absolute number of recent thymic emigrants in peripheral blood and in the thymus. **(A,B)** Absolute count of Recent Thymic Emigrants in the thymus (HD, *n* = 26; DGS, *n* = 4; DS, *n* = 8) **(A)** and in the peripheral blood (HD, *n* = 34; DGS, *n* = 10; DS, *n* = 8) **(B)**; mean ± SEM are represented (Mann-Whitney test; ^*^*p*-value < 0.01).

Supplemental Table 1 Healthy Donor's clinical data. We received and analyzed thymic tissue recovered from all HDs listed in the table; CHD, congenital heart defect; NA, not available; TOF, tetralogy of Fallot; ASD, atrial septal defect; APW, aortopulmonary window; CoA, aortic coarctation; AI, aortic incompetence; VSD, ventricular septal defect; DORV, double-outlet right ventricle; HLHS, hypoplastic left heart syndrome; PAVC, partial atrioventricular canal defect.

Supplemental Table 2 Patient's clinical data. ^*^Analysis of thymic tissue; RTI, respiratory tract infection; ARDS, acute respiratory distress syndrome; JIA, juvenile idiopathic arthritis; ANA, anti-nuclear antibody, CHD, congenital heart defect; NA, not available; TOF, tetralogy of Fallot; ASD, atrial septal defect; VSD, ventricular septal defect; DORV, double-outlet right ventricle; PAVC, partial atrioventricular canal defect; NCS, not cardiac surgery.

Supplemental Table 3 Number of patients evaluated for thymocyte and peripheral blood immunophenotype.
