# Supplementary figures and images for: Thymic Epithelium Abnormalities in DiGeorge and Down Syndrome Patients Contribute to Dysregulation in T Cell Development
Source: Front Immunol. 2019 Mar 15;10:447. doi: 10.3389/fimmu.2019.00447 (PMC6436073; doi:10.3389/fimmu.2019.00447)

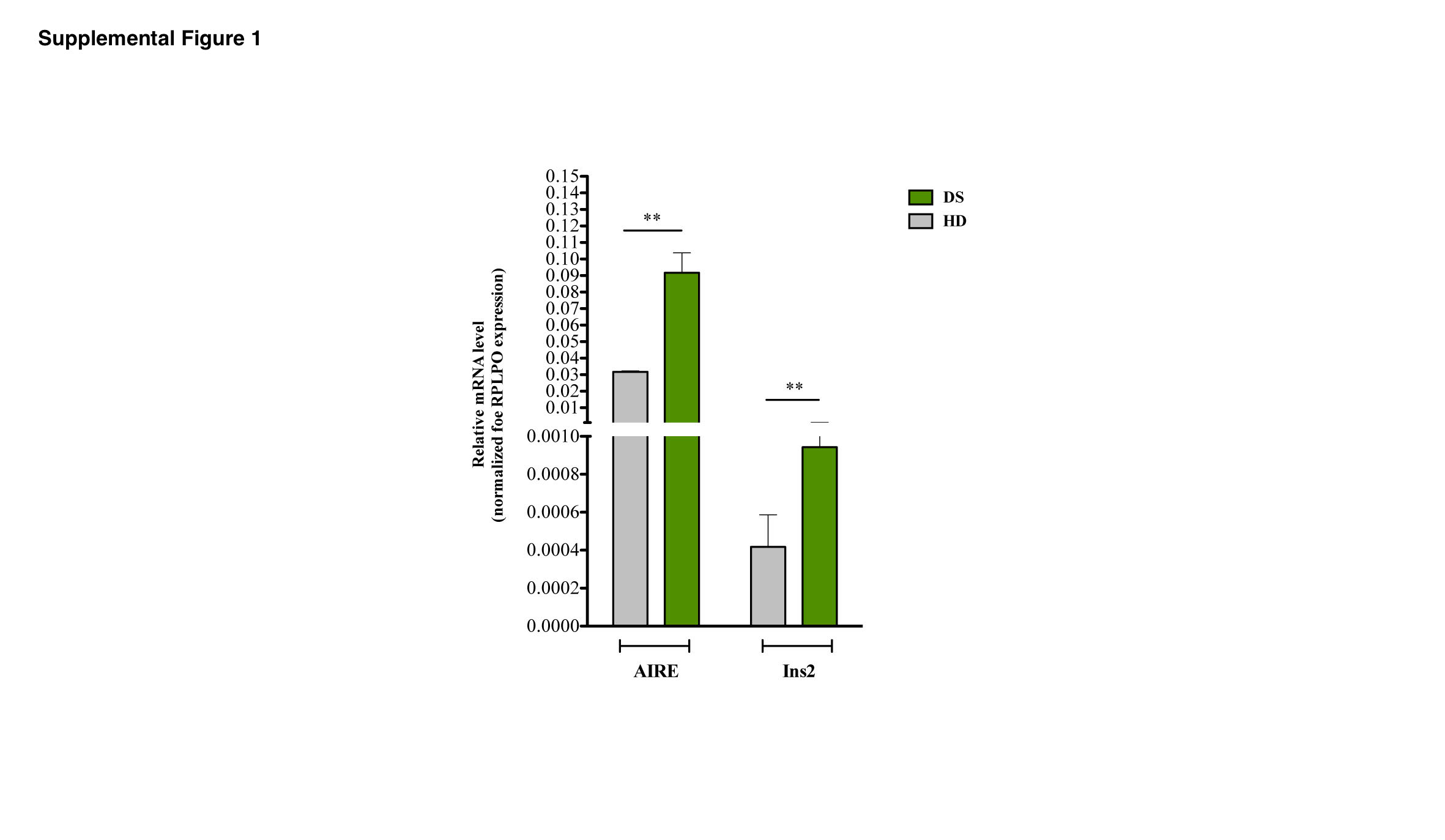

Supplement: Supplemental Figure 1 — Increased AIRE and Ins2 expression in thymic tissue from DS patients. Comparison of mRNA expression of AIRE and tissue restricted antigen AIRE-dependent, Ins2, in HDs and DS patients, normalized for the expression of the housekeeping gene RPLPO (HD, n = 3; DS, n = 3). Mean ± SEM are represented (Two-way ANOVA test; **p-value < 0.002). [file Image_1.JPEG]

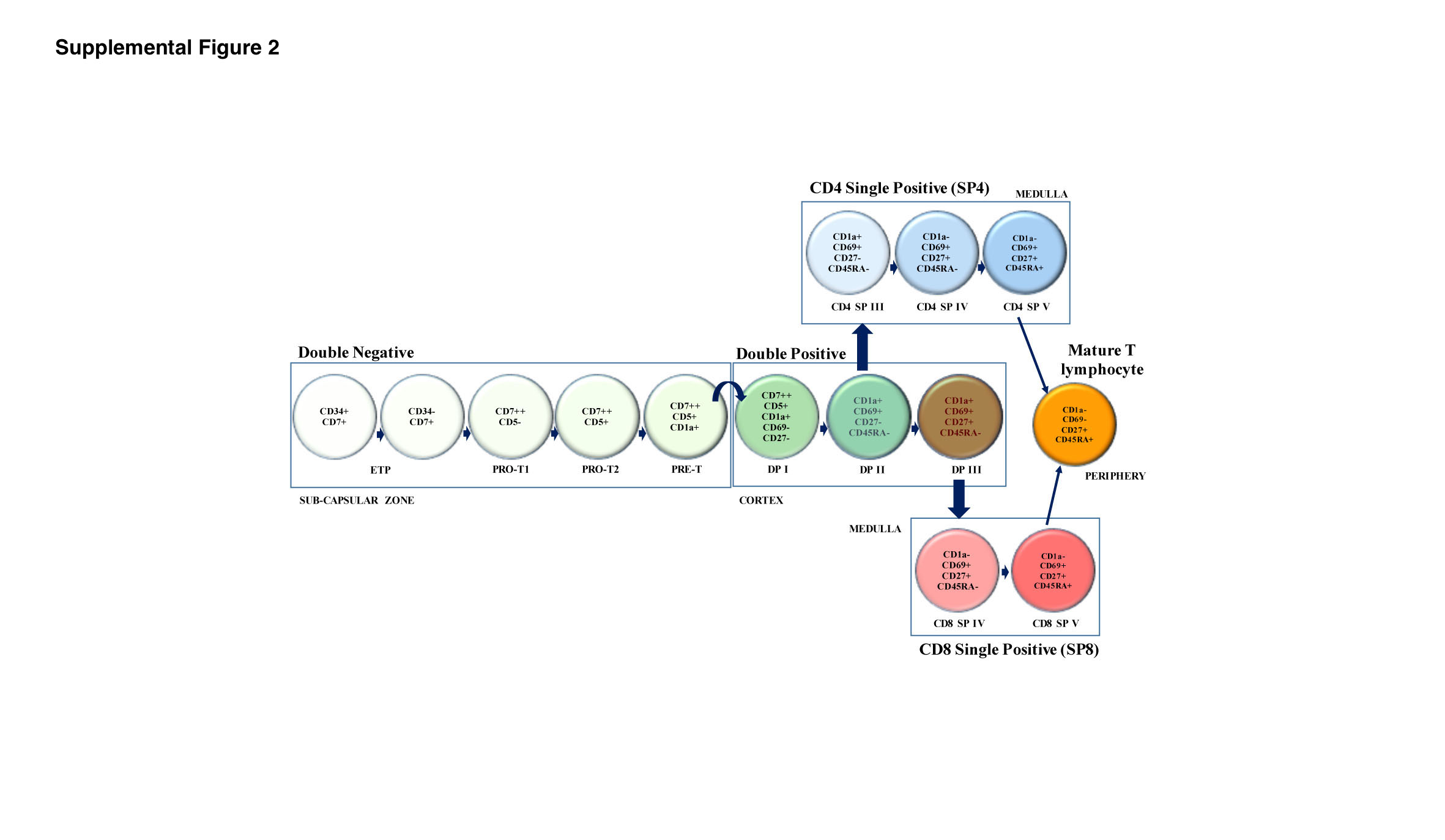

Supplement: Supplemental Figure 2 — Thymocyte maturational stages from double negative stage to mature T lymphocytes. Sequential view of the different phenotypical markers expressed during thymocyte differentiation and thymic emigration of CD4+ and CD8+ T cells. Different maturational stages can be defined by the relative expression of specific markers, such as CD69, CD27, CD7, and CD1a [modified from Vanhecke et al. (27); Reimann et al. (28)]. [file Image_2.JPEG]

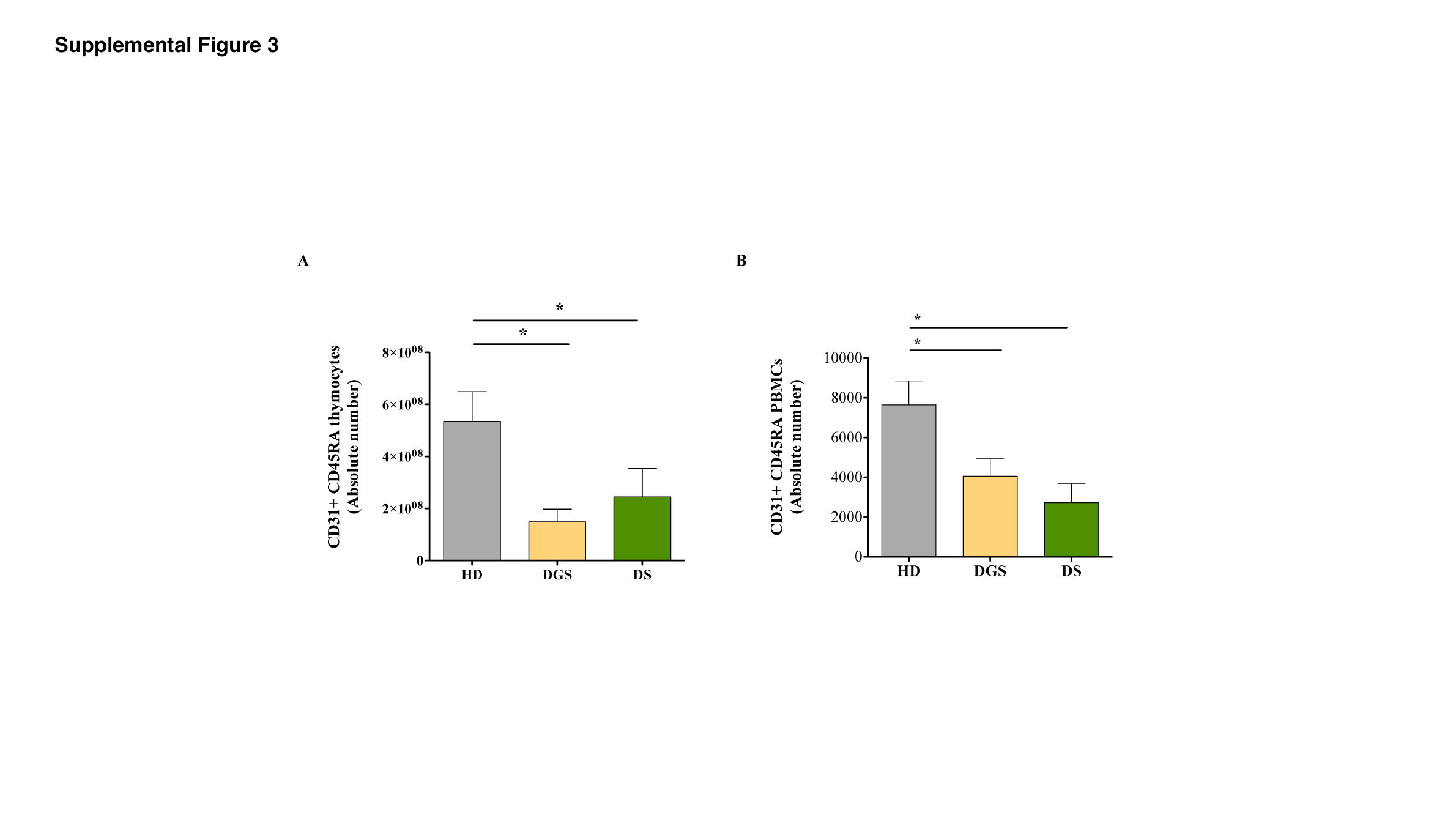

Supplement: Supplemental Figure 3 — Absolute number of recent thymic emigrants in peripheral blood and in the thymus. (A,B) Absolute count of Recent Thymic Emigrants in the thymus (HD, n = 26; DGS, n = 4; DS, n = 8) (A) and in the peripheral blood (HD, n = 34; DGS, n = 10; DS, n = 8) (B); mean ± SEM are represented (Mann-Whitney test; *p-value < 0.01). [file Image_3.JPEG]

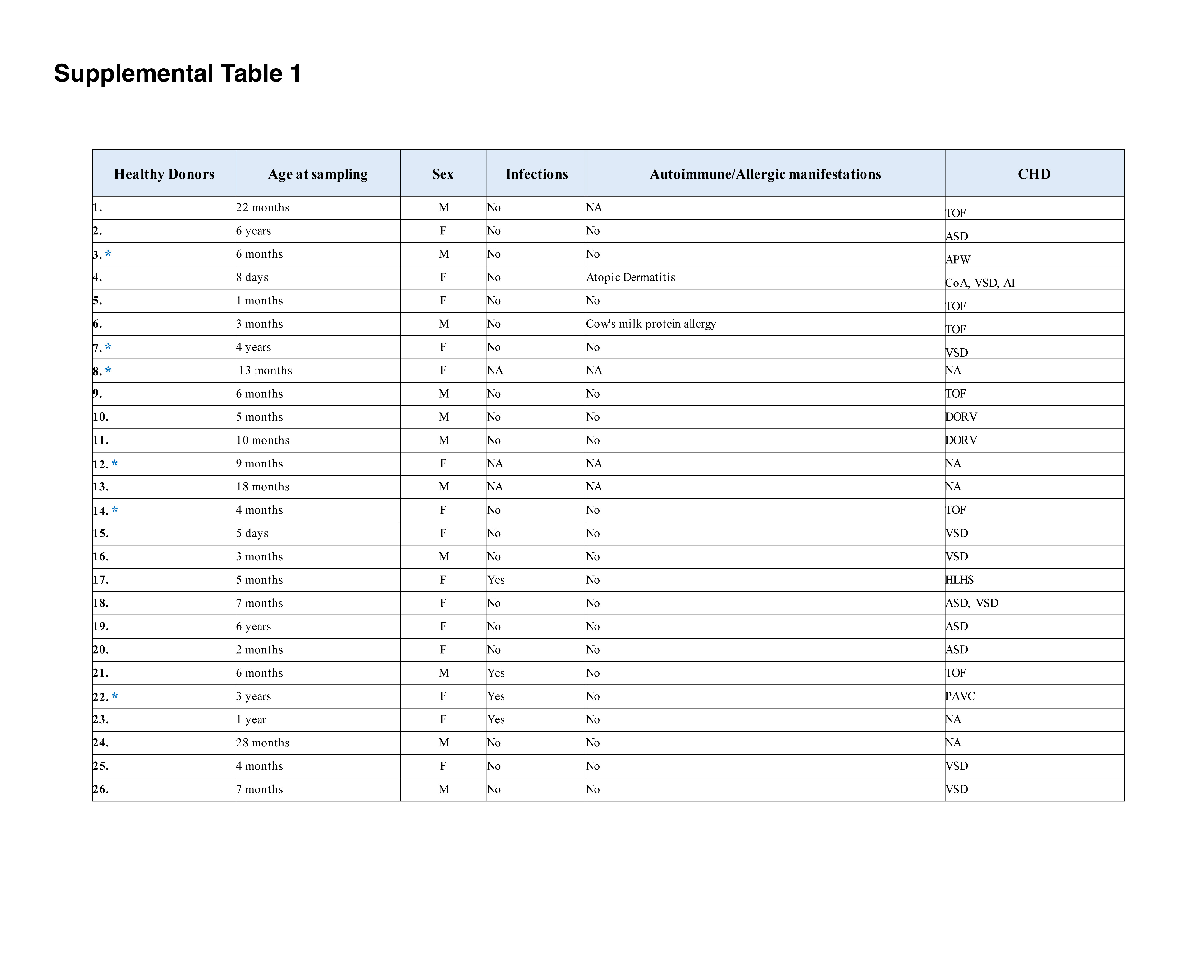

Supplement: Supplemental Table 1 — Healthy Donor's clinical data. We received and analyzed thymic tissue recovered from all HDs listed in the table; CHD, congenital heart defect; NA, not available; TOF, tetralogy of Fallot; ASD, atrial septal defect; APW, aortopulmonary window; CoA, aortic coarctation; AI, aortic incompetence; VSD, ventricular septal defect; DORV, double-outlet right ventricle; HLHS, hypoplastic left heart syndrome; PAVC, partial atrioventricular canal defect. [file Image_4.JPEG]

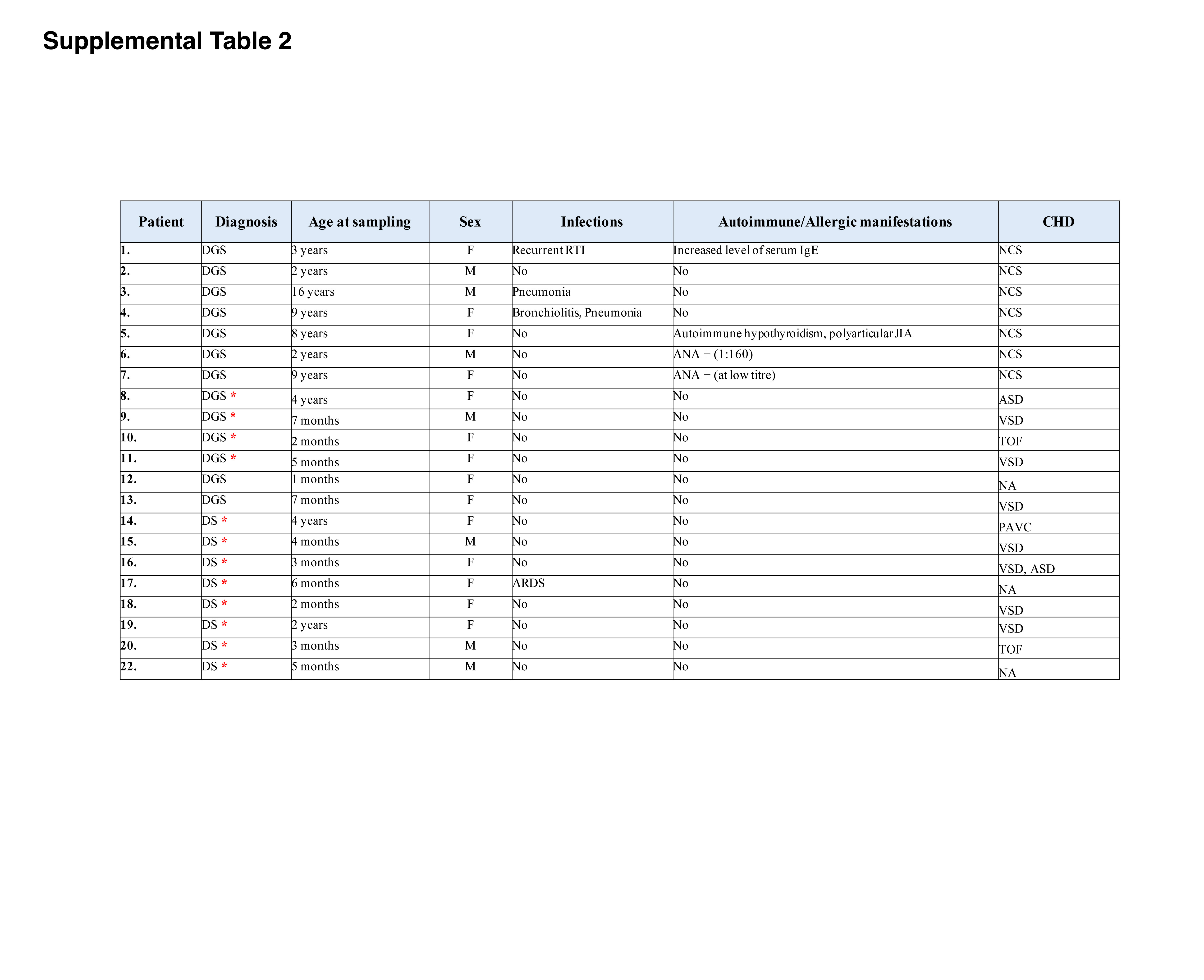

Supplement: Supplemental Table 2 — Patient's clinical data. *Analysis of thymic tissue; RTI, respiratory tract infection; ARDS, acute respiratory distress syndrome; JIA, juvenile idiopathic arthritis; ANA, anti-nuclear antibody, CHD, congenital heart defect; NA, not available; TOF, tetralogy of Fallot; ASD, atrial septal defect; VSD, ventricular septal defect; DORV, double-outlet right ventricle; PAVC, partial atrioventricular canal defect; NCS, not cardiac surgery. [file Image_5.JPEG]

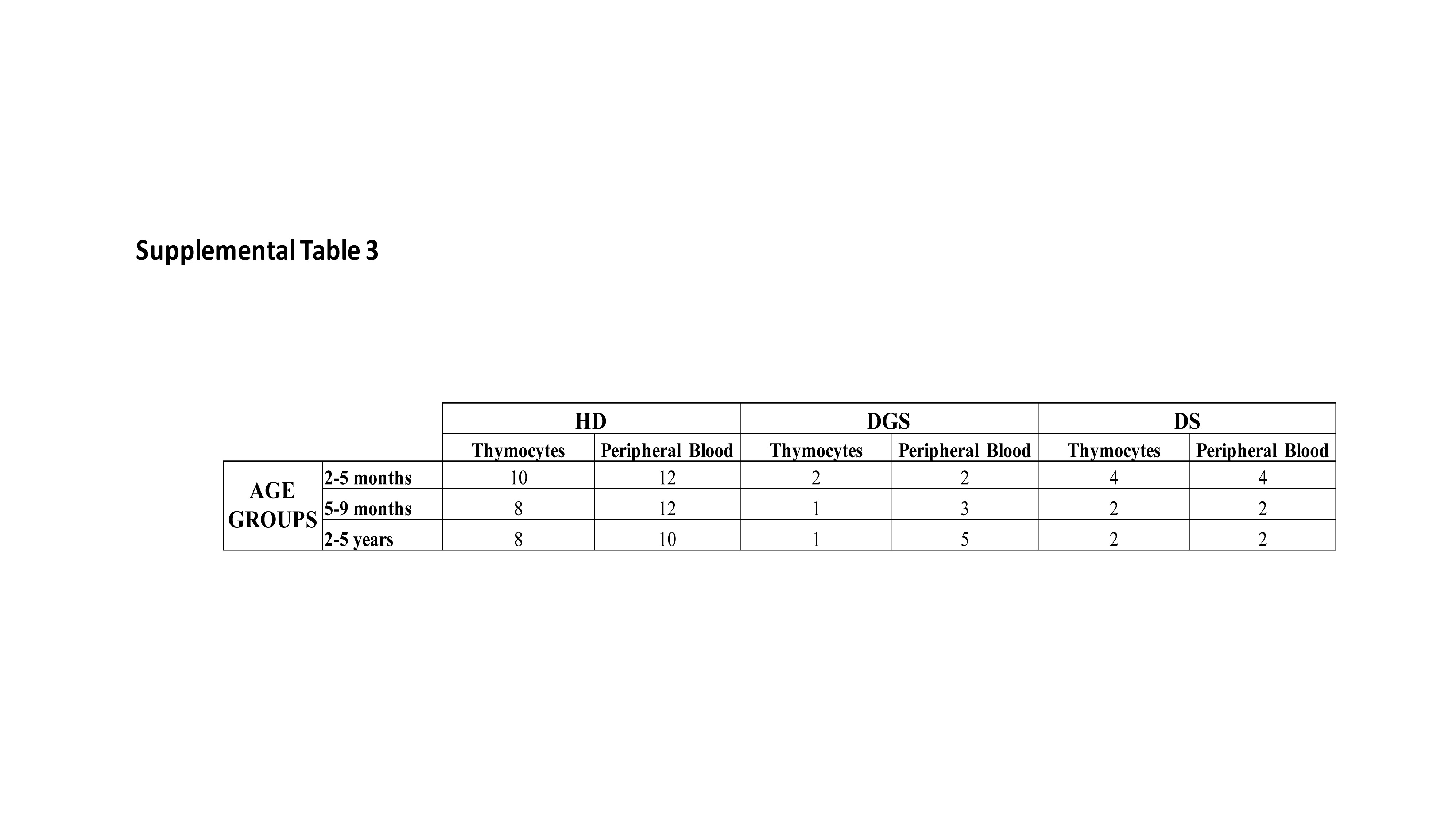

Supplement: Supplemental Table 3 — Number of patients evaluated for thymocyte and peripheral blood immunophenotype. [file Image_6.JPEG]
